# Spatial frequency supports the emergence of categorical representations in visual cortex during natural scene perception

**DOI:** 10.1016/j.neuroimage.2018.06.033

**Published:** 2018-10-01

**Authors:** Diana C. Dima, Gavin Perry, Krish D. Singh

**Affiliations:** Cardiff University Brain Research Imaging Centre (CUBRIC), School of Psychology, Cardiff University, Cardiff, CF24 4HQ, United Kingdom

**Keywords:** Multivariate pattern analysis (MVPA), Representational similarity analysis (RSA), Magnetoencephalography (MEG), Convolutional neural network (CNN), Scene categorization

## Abstract

In navigating our environment, we rapidly process and extract meaning from visual cues. However, the relationship between visual features and categorical representations in natural scene perception is still not well understood. Here, we used natural scene stimuli from different categories and filtered at different spatial frequencies to address this question in a passive viewing paradigm. Using representational similarity analysis (RSA) and cross-decoding of magnetoencephalography (MEG) data, we show that categorical representations emerge in human visual cortex at ∼180 ms and are linked to spatial frequency processing. Furthermore, dorsal and ventral stream areas reveal temporally and spatially overlapping representations of low and high-level layer activations extracted from a feedforward neural network. Our results suggest that neural patterns from extrastriate visual cortex switch from low-level to categorical representations within 200 ms, highlighting the rapid cascade of processing stages essential in human visual perception.

## Highlights

• Low spatial frequencies support generalizable neural responses to natural scenes.
• Categorical representations of scenes arise in visual cortex within 200 ms.
• We find simultaneous processing of low- and high-level visual features.
• Low- and high-level neural network layers have overlapping representations.

## Introduction

Classic models of natural vision entail a hierarchical process transforming low-level properties into categorical representations (VanRullen and Thorpe, 2001; Yamins and DiCarlo, 2016). During early stages of natural scene perception, the primary visual cortex processes low-level stimulus properties using inputs from the retina via the lateral geniculate nucleus (Hubel and Wiesel, 1962). Extrastriate and scene-selective areas are associated with mid-level and high-level properties, with categorical, invariant representations considered the final stage of abstraction (Felleman and Van Essen, 1991; Ungerleider and Haxby, 1994). Scene-selective brain regions such as the parahippocampal place area (PPA), the retrosplenial cortex (RSC), and the occipital place area (OPA) are often thought to represent such categories (Walther et al., 2009) and have been found to respond to high-level stimuli in controlled experiments (Schindler and Bartels, 2016; Walther et al., 2011).

However, this model has been challenged by evidence of low- and mid-level features being processed in scene-selective areas (Kauffmann et al., 2015b; Kravitz et al., 2011; Nasr et al., 2014; Nasr and Tootell, 2012; Rajimehr et al., 2011; Watson et al., 2014, 2016). Studies of temporal dynamics have found overlapping signatures of low-level and high-level representations (Groen et al., 2013; Harel et al., 2016), suggesting co-occurring and co-localized visual and categorical processing (Ramkumar et al., 2016). Such evidence casts doubt on the hierarchical model and on the usefulness of the distinction between low-level and high-level properties (Groen et al., 2017).

In particular, spatial frequency is thought to play an important part in natural scene perception, with low spatial frequencies mediating an initial rapid parsing of visual features in a “coarse-to-fine” sequence (Kauffmann et al., 2015b). Its role in the processing speed of different features, as well as evidence of its contribution to neural responses in scene-selective areas (Rajimehr et al., 2011), makes spatial frequency a particularly suitable candidate feature for teasing apart the temporal dynamics of low and high-level natural scene processing.

Recent neuroimaging studies of scene perception have used multivariate pattern analysis (MVPA) to highlight the links between low-level processing and behavioural goals (Ramkumar et al., 2016; Watson et al., 2014). In particular, Ramkumar et al. (2016) showed successful decoding of scene gist from MEG data and linked decoding performance to spatial envelope properties, as well as behaviour in a categorization task.

In the present study, we aimed to dissociate the role of low-level and high-level properties in natural scene perception, in the absence of behavioural goals that may influence visual processing (Groen et al., 2017). In order to do so, we recorded MEG data while participants passively viewed a controlled stimulus set composed of scenes and scrambled stimuli filtered at different spatial frequencies. Thus, we were able to first contrast responses to scenes with responses to matched control stimuli (which, to the extent of our knowledge, have not yet been used in the M/EEG literature on natural scenes); and second, we were able to assess the presence of a categorical response to scenes invariant to spatial frequency manipulations.

We used multivariate pattern analysis (MVPA) and representational similarity analysis (RSA) to explore representations of scene category in space and time and to assess their relationship to low-level properties. Multivariate analyses are sensitive to differences in overlapping patterns (Norman et al., 2006) and can describe the spatiotemporal dynamics and structure of neural representations through information mapping (Kriegeskorte et al., 2008, 2006).

We successfully decoded scene category from MEG responses in the absence of an explicit categorization task, and a cross-decoding analysis suggested that this effect is driven by low spatial frequency features at ∼170 ms post-stimulus onset. We also show that categorical representations arise in extrastriate visual cortex within 200 ms, while at the same time representations in posterior cingulate cortex correlate with the high-level layers of a convolutional neural network. Together, our results suggest that scene perception relies on low spatial frequency features to create an early categorical representation in visual cortex.

## Methods

### Participants

Nineteen participants took part in the MEG experiment (10 females, mean age 27, standard deviation SD 4.8), and fourteen in a control behavioural experiment (13 females, mean age 26, SD 4.4). All participants were healthy, right-handed and had normal or corrected-to-normal vision (based on self-report). Written consent was obtained in accordance with The Code of Ethics of the World Medical Association (Declaration of Helsinki). All procedures were approved by the ethics committee of the School of Psychology, Cardiff University.

### Stimuli

Stimuli (Supplementary Figure 2) were 20 natural scenes (fields, mountains, forests, lakes and seascapes) and 20 urban scenes (office buildings, houses, city skylines and street views) from the SUN database (Xiao et al., 2010). Stimuli were 800 × 600 pixels in size, subtending 8.6 × 6.4 degrees of visual angle.

All the images were converted to grayscale. Using the SHINE toolbox (Willenbockel et al., 2010), luminance and contrast were normalized to the mean luminance and SD of the image set. Spatial frequency was matched across stimuli by equating the rotational average of the Fourier amplitude spectra (the energy at each spatial frequency across orientations).

To assess the similarity of image amplitude spectra between categories, we calculated pairwise Pearson's correlation coefficients based on pixel intensity values between all images (mean correlation coefficient 0.14, SD 0.27, minimum-maximum range 1.33). Next, we performed an equivalence test (two one-sided tests; Lakens, 2017) in order to compare within-category correlation coefficients from both conditions (i.e., pairwise correlation coefficients between each image and each of the 19 images belonging to the same category) to between-category correlation coefficients (i.e., pairwise correlation coeffients between each image and each of the 20 images belonging to the other category). We assumed correlation coefficients to be similar if the difference between them fell within the [-0.1, 0.1] equivalence interval (Cohen, 1992). Within-category and between-category correlation coefficients were found to be equivalent (*P*_*1*_ = 5.3*10^−11^, *P*_*2*_ = 2.4*10^−4^, 90% CI [-0.0025, 0.063]).

Prior to spatial frequency filtering, the mean of each image was set to 0 to avoid DC artefacts and effects induced by zero-padding. To obtain low spatial frequency (LSF) and high spatial frequency (HSF) stimuli, we applied a low-pass Gaussian filter with a cutoff frequency of 3 cycles per degree (25.8 cycles per image) and a high-pass filter with a cutoff of 6 cycles per degree (51.6 cycles per image). Root mean square (RMS) contrast (standard deviation of pixel intensities divided by their mean) was only normalized within and not across spatial frequency conditions, in order to maintain the characteristic contrast distribution typical of natural scenes, which has been shown to influence responses to spatial frequency in the visual system (Field, 1987; Kauffmann et al., 2015b, 2015a).

**Fig. 1:**
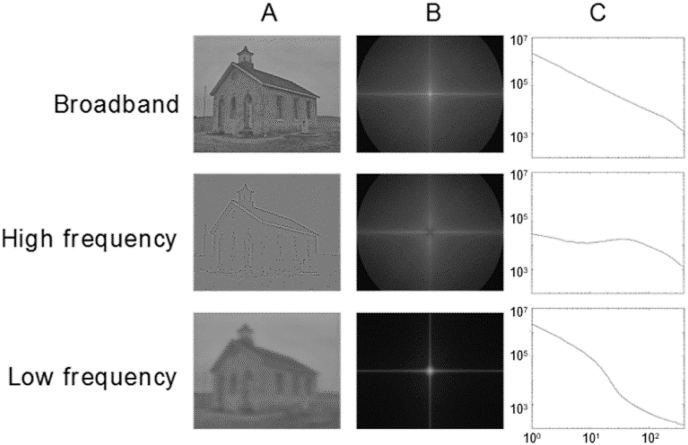
Examples of urban scene stimuli filtered at different spatial frequencies (A), together with the average Fourier spectra (B) and frequency power spectra (C) for each stimulus set (log spectral power on the y-axis plotted against log spatial frequency on x-axis).

To produce control stimuli, we scrambled the phase of the images in the Fourier domain, ensuring equivalent Fourier amplitude spectra across the original and scrambled images (Perry and Singh, 2014). For each spatial frequency condition, we randomly selected 10 of the 20 phase-scrambled images for use in the experiment in order to maintain an equal number of stimuli across conditions (natural, urban and scrambled). The final stimulus set contained 180 images (filtered and unfiltered scenes and scrambled stimuli; Fig. 1, Supplementary Figure 1).

### Behavioural experiment

#### Design and data collection

To assess potential differences in the recognizability of different scenes, participants in the behavioural experiment viewed the stimuli and were asked to categorize them as fast as possible. The design of the behavioural experiment was similar to the MEG experiment, but included a practice phase (10 trials) before each block. Participants underwent two blocks in which they had to judge whether stimuli were scenes or scrambled stimuli, or whether scene stimuli were natural or urban respectively. Blocks were separated by a few minutes' break and their order was counterbalanced across subjects.

Images were presented on an LCD monitor with a resolution of 1920 × 1080 pixels and a refresh rate of 60 Hz. Participants were required to make a keyboard response (using the keys ‘J’ and ‘K’, whose meanings were counterbalanced across subjects), as soon as each image appeared on screen. We recorded responses and reaction times using Matlab R2015a (The Mathworks, Natick, MA, USA) and the Psychophysics Toolbox (Brainard, 1997; Pelli, 1997; Kleiner et al., 2007).

#### Data analysis

To assess the effect of spatial frequency filtering on performance in the categorization task, one-way repeated-measures ANOVAs were performed on individual accuracies (after performing a rationalized arcsine transformation; Studebaker, 1985) and on mean log-transformed reaction times for each categorization task (four tests with a Bonferroni-adjusted alpha of 0.0125). Significant effects were followed up with post-hoc Bonferroni-corrected paired t-tests.

### MEG data acquisition

For source reconstruction purposes, in all participants, we acquired whole-head structural MRI scans on a General Electric 3 T MRI scanner using a 1 mm isotropic Fast Spoiled Gradient-Recalled-Echo pulse sequence in an oblique-axial orientation, with a field of view of 256 × 192 × 176 mm (TR/TE = 7.9/3.0 ms, inversion time = 450 ms, flip angle = 20°).

Whole-head MEG recordings were made using a 275-channel CTF radial gradiometer system at a sampling rate of 1200 Hz. Three of the sensors were turned off due to excessive sensor noise. An additional 29 reference channels were recorded for noise rejection purposes; this allowed the primary sensors to be analysed as synthetic third-order gradiometers using a linear combination of the weighted reference sensors (Vrba and Robinson, 2001).

Stimuli were centrally presented on a grey background using a gamma-corrected Mitsubishu Diamond Pro 2070 CRT monitor with a refresh rate of 100 Hz and a screen resolution of 1024 × 768 pixels situated at a distance of 2.1 m from the participants. There were 9 conditions (natural scenes, urban scenes and scrambled scenes filtered at low frequency, high frequency or unfiltered). Each image was presented 4 times, amounting to 80 trials per condition. Participants underwent two recording sessions separated by a few minutes' break.

The data were collected in 2.5 s epochs centred around the stimulus onset. Stimuli were presented on screen for 1 s and were followed by a fixation cross for a varying ISI chosen pseudorandomly from a uniform distribution between 0.6 and 0.9 s. Participants were instructed to press a button whenever the fixation cross changed colour during the ISI. The paradigm was implemented using Matlab R2015a and the Psychophysics Toolbox.

Participants were seated upright while viewing the stimuli and electromagnetic coils were attached to the nasion and pre-auricular points on the scalp in order to continuously monitor head position relative to a fixed coordinate system on the dewar. We acquired high-resolution digital photographs to verify the locations of the fiducial coils and co-register them with the participants' structural MRI scans. An SMI iView X eyetracker system with a sampling rate of 250 Hz was used to track the subjects' right pupil and corneal reflection while viewing the stimuli.

### MEG decoding analyses

The data were pre-processed using Matlab R2015a and the FieldTrip toolbox (Oostenveld et al., 2011). Trials containing excessive eye or muscle-related artefacts were excluded based on visual inspection. Although using an automatic artefact rejection algorithm would be preferable in order to reduce subjectivity (e.g. Jas et al., 2017; Nolan et al., 2010), we note that condition information was not available during artefact rejection, and there was no significant difference in the proportion of trials rejected between conditions (*P* > 0.06, 3 × 3 ANOVA). To account for head motion, we excluded trials with maximum motion of any individual fiducial coil in excess of 5 mm. We quantified motion as the maximum displacement (change in position between sample points) of the fiducial coils during any given trial. To account for potential changes in the participants' head position over time, head coil position relative to the dewar was changed to the average position across all trials.

Prior to sensor-space MVPA analyses, the data were resampled to 600 Hz and bandpass-filtered between 0.5 and 100 Hz. A 50 Hz comb filter was used to remove the mains noise and its harmonics. Baseline correction was applied using a time window of 500 ms prior to the stimulus onset.

To test for differences between conditions present in single trials, a linear Support Vector Machine (SVM) classifier was applied to sensor-level data. The classifier was implemented in Matlab using the Statistics and Machine Learning Toolbox and the Bioinformatics Toolbox. SVM is robust to high-dimensional feature vectors due to its in-built regularization (Nilsson et al., 2006), while the choice of a linear kernel improves the interpretability of classification results (Ritchie and Carlson, 2016).

#### Decoding responses to unfiltered scenes

##### Sensor-space MVPA

**Fig. 2:**
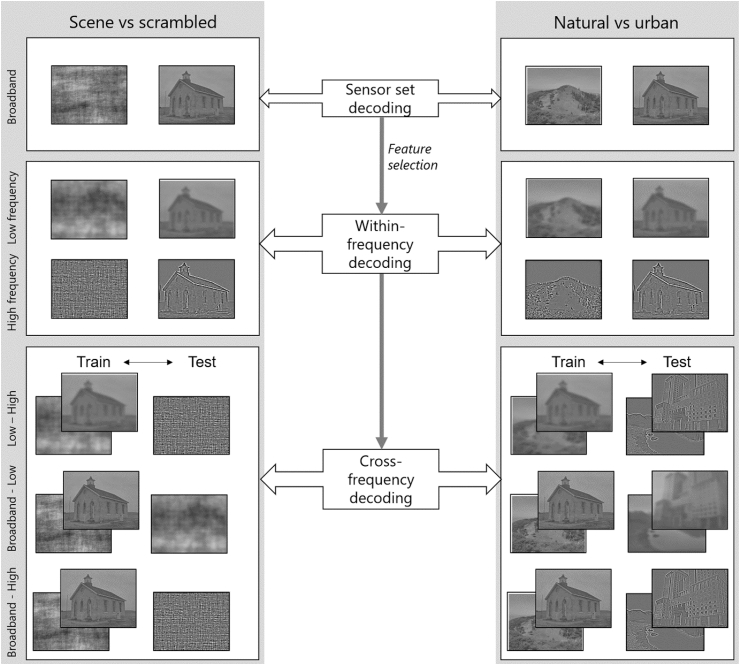
The sensor-space MVPA analysis pipeline. Note that in cross-decoding each stimulus set acted in turns as a training and test set and resulting accuracies were averaged across the two cases. Cross-exemplar five-fold cross-validation was performed for all analyses.

A first MVPA analysis (Fig. 2) was performed on responses to unfiltered stimuli using single-trial data from four anatomically defined sensor sets (occipital, temporal, parietal and fronto-central; Fig. 4). Binary time-resolved classification was applied to broadband scenes and scrambled stimuli, as well as broadband natural and urban scenes. As the former problem entailed unequal class sizes, majority class trials were randomly sub-sampled.

The classifier was applied to each time point between 0.5 s pre-stimulus onset and 1 s post-stimulus onset after resampling the data to 600 Hz, thus giving a temporal resolution of ∼1.6 ms. Feature vectors were standardized using the mean and standard deviation of the training set. To evaluate classifier performance within subjects, we used cross-exemplar five-fold cross-validation, whereby the classifier was iteratively trained on trials corresponding to 16 of the 20 stimuli from each condition and tested on the remaining 4 stimuli. This ensured that classification performance was not driven by responses to particular visual features repeated across the training and test sets, whilst achieving balanced training and test sets and reducing variability in classification performance.

An additional sensor-level searchlight decoding analysis was performed, which is reported in the Supplementary Material (Supplementary Analysis 2).

##### Source-space MVPA

To perform classification in source space, data in all trials regardless of condition were bandpass-filtered between 0.5 and 100 Hz. We used the FSL Brain Extraction Tool (Smith, 2002) to extract the brain surface from the participants' structural MRI scans and we projected the data into source space using the Linearly Constrained Minimum Variance (LCMV) beamformer (Van Veen et al., 1997). LCMV constructs an adaptive spatial filter by combining the forward model (here, a single-shell sphere) and the data covariance matrix (Hillebrand et al., 2005).

We defined the source space using a template grid with a resolution of 10 mm that was warped to each participant's MRI in order to ensure equivalence of sources across participants. For each voxel, we independently derived the output as a weighted sum of all MEG sensor signals. The beamformer algorithm entails no assumptions about the number of active sources and has the additional advantage of suppressing eye movement artefacts (Kinsey et al., 2011).

The decoding analysis was performed using an anatomically informed searchlight approach based on the Automated Anatomical Labeling (AAL) atlas (Tzourio-Mazoyer et al., 2002). For each subject, time-resolved classification with cross-exemplar cross-validation as described above (section 2.4) was performed iteratively using the timecourses of sources from each AAL region of interest (ROI), excluding the cerebellum and some deep structures. We chose this approach to reduce computational cost, to improve interpretability across studies and modalities (Hillebrand et al., 2012), and to overcome some of the caveats of traditional searchlight analyses, which assume that information is uniformly distributed in the brain (Etzel et al., 2013).

#### Using MVPA to evaluate the role of spatial frequency

To maximize the amount of informative features input to the classifier, we performed the next MVPA analyses using the occipital sensor set, which achieved the best classification performance in the broadband scene vs scrambled decoding problem. This ensured minimal overlap between the decoding problem used in feature selection and the follow-up analyses (Fig. 2).

##### Decoding responses to filtered stimuli

Despite the use of matched control stimuli, successful decoding of unfiltered scenes does not allow us to disentangle low-level and high-level responses, as differences in local low-level properties cannot be ruled out. Thus, to assess the role played by spatial frequency, we performed scene category decoding (scenes vs scrambled stimuli and natural vs urban scenes) within each spatial frequency condition (HSF and LSF) using the occipital sensor set and cross-exemplar cross-validation.

##### Cross-decoding

Next, we aimed to test whether scene category representations generalized across spatial frequency categories. To this aim, we trained and tested sensor-space scene category classifiers across different spatial frequency conditions. The analysis was repeated for all three condition pairs using five-fold cross-exemplar cross-validation, with each set of stimuli acting as a training set and as a test set in turns and the final accuracy averaged across the two cases (Fig. 2).

In this analysis, classifier performance was interpreted as an index of the similarity of scene-specific responses across spatial frequency manipulations. Successful decoding across LSF and HSF stimuli would indicate a truly spatial frequency-independent categorical distinction, as there are no overlapping spatial frequencies across the two sets. On the other hand, cross-decoding across unfiltered and LSF or HSF scenes would allow us to detect any spatial frequency preference in the encoding of scene-specific information.

The fact that RMS contrast was not normalized across spatial frequency conditions introduced a potential confound in this analysis. This was not an issue when training and testing within one spatial frequency condition (as RMS contrast was normalized across stimulus categories within each spatial frequency condition). However, both local and global amplitude characteristics were similar between broadband and LSF scenes due to the 1/*f* amplitude spectrum of natural scenes discussed above; this posed a specific concern to the cross-decoding of broadband and LSF scenes. This issue was addressed by conducting cross-exemplar cross-validation. Normalization of low-level features within training and test sets ensured that global contrast characteristics would not be exploited in classification, while testing on novel exemplars ensured that the classifier would not simply “recognize” local features (including contrast) unaffected by the spatial frequency manipulation. This does not preclude the existence of local characteristics that distinguish scenes from scrambled stimuli; however, such characteristics can be expected to be informative in the emergence of a high-level response.

#### Significance testing

Averaged accuracy across subjects (proportion correctly classified trials) was used to quantify decoding performance, and the significance of classifier accuracy was assessed through randomization testing (Nichols and Holmes, 2001; Noirhomme et al., 2014). As accuracies can sometimes rise above the theoretical chance level as an artefact of small sample sizes (Jamalabadi et al., 2016), estimating an empirical chance level offers a robust method of assessing classification performance.

To this end, 1000 randomization iterations were performed for each subject, whereby class labels were shuffled across the training and test sets before recomputing classification accuracy. The null distribution was estimated based on the time point achieving maximum overall accuracy in the MVPA analysis. For time-resolved sensor-space decoding analyses, P-values (α = 0.01) were omnibus-corrected using the maximum accuracy across all tests performed (Nichols and Holmes, 2001; Singh et al., 2003), and cluster-corrected across time. To determine 95% confidence intervals around decoding onset latencies, individual decoding accuracies were bootstrapped 1000 times with replacement, and differences in onset latencies were tested using a Wilcoxon signed-rank test. For searchlight decoding in sensor and source space, P-values (α = 0.001) were thresholded using the maximum accuracy across sensor clusters/ROIs and cluster-corrected across time.

### Representational similarity analysis (RSA)

While MVPA offers a measure of latent category-specific information available in neural data (Kriegeskorte et al., 2006), it does not provide evidence about the type of representation underpinning successful decoding. Previous studies have shown that similarity-based measures can tease apart different types of representations underlying spatiotemporal neural patterns (Kriegeskorte et al., 2008; Kriegeskorte and Kievit, 2013). In order to evaluate low and high-level representations of stimuli in our data, we assessed correlations between representational dissimilarity matrices (RDMs) based on temporally and spatially resolved MEG patterns and two sets of models: (1) explicit feature-based models (based on either stimulus properties or stimulus categories), and (2) models extracted from the layers of a CNN. The second analysis was performed to assess whether evaluating an explicitly hierarchical set of models would support our initial conclusions.

#### Feature-based models

**Fig. 3:**
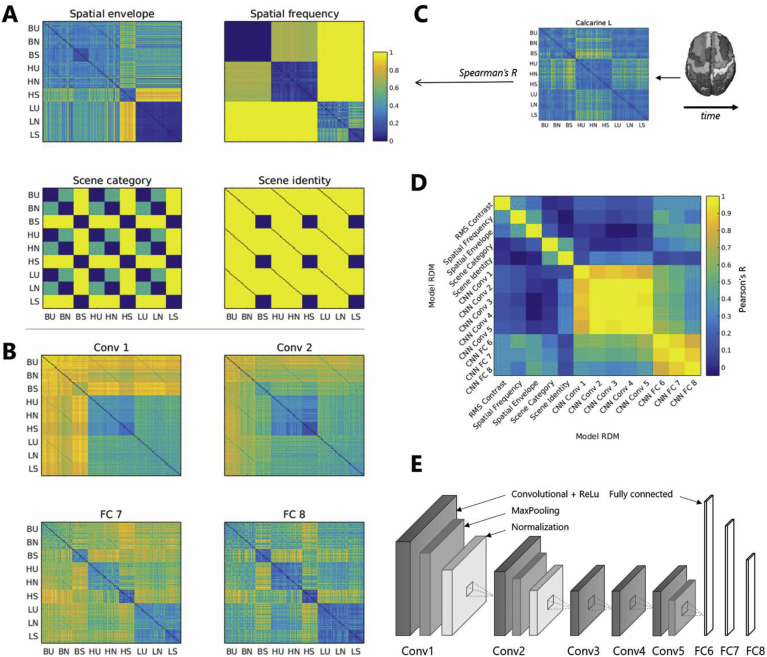
**A.** Feature-based model RDMS. Values of 0 represent maximal similarity according to the model, while values of 1 represent maximal distance. Stimulus sets are denoted by two letters representing spatial frequency condition (B: broadband, H: high frequency, L: low frequency) and category (U: urban, N: natural, S: scrambled). **B.** Examples of CNN-based model RDMs. Conv: convolutional layer; FC: fully-connected layer. **C.** Representation of the RSA analysis framework: time-resolved neural RDMS were estimated for each ROI and correlated with the model RDMs using Spearman's partial correlation. **D.** Correlations between all model RDMs. RDMs based on convolutional layers and fully connected layers of the CNN are highly correlated. **E.** The CNN architecture used for model RDM generation.

In order to assess the contributions of low-level features and categorical distinctions, we first evaluated four model RDMs of stimulus representation (Fig. 3). Two models based on visual stimulus features were tested: a low-level model based on spatial frequency, and a mid-level model reflecting the spatial envelope of the images. The former was based on pairwise Euclidean distances between the spatial frequency spectra of the images; the latter was computed using the GIST descriptor (Oliva et al., 2001), which applies a series of Gabor filters at different orientations and positions in order to extract 512 values for each image. These values represent the average orientation energy at each spatial frequency and position and were used to compute pairwise Euclidean distances.

For high-level representations, we used a category-based and an identity-based model. In the former model, all scenes within a category (such as urban scenes) were assigned a distance of 0, while scrambled stimuli and scenes were assigned a maximal distance of 1, and distances between different categories of scenes (natural and urban) were set to 0.5. The scene identity model assigned dissimilarity values of 1 to all pairs of natural scenes regardless of category (while all scrambled stimuli were deemed maximally similar). For both models, these values were constant across spatial frequency manipulations.

#### CNN-based models

To more directly assess the hierarchical processing of our stimulus set in the visual system, we tested a second set of models derived from the layers of a feedforward CNN. Using MATLAB R2017a and the Neural Network Toolbox, we extracted features from an eight-layer CNN pre-trained using the Caffe framework (Jia et al., 2014; http://caffe.berkeley-vision.org/) on the Places database, which consists of 2.5 million images from 205 scene categories (Zhou et al., 2014). The neural network was a well-established AlexNet CNN (Krizhevsky et al., 2012) with five convolutional layers and three fully-connected layers. This network architecture has been shown to perform well in explaining object and scene representations in the visual system (e.g. Cichy et al., 2016b; Rajaei et al., 2018). We extracted network activations from the last stage of each CNN layer for each image in our stimulus set, and we calculated pairwise Euclidean distances between the feature vectors to obtain eight CNN-based RDMs (Fig. 3). To assess how well scene categories were represented by these features, we also performed cross-validated binary classification (unfiltered scene vs scrambled and urban vs natural images) using layer activations, and found high decoding accuracies in all layers (>70%; Supplementary Figure 4).

#### RSA analysis framework

In order to assess correlations between model RDMs and neural patterns, MEG data were pre-processed and projected into source space as described above. Neural patterns were computed using source timecourses within each AAL-based ROI for each 16 ms time window after stimulus onset in order to decrease computational cost. Responses to repeated stimuli were averaged within and across subjects and the Euclidean distance between each pair of stimuli was computed to create neural RDMs.

For each ROI and time window, we computed Spearman's rank partial correlation coefficients between the neural dissimilarity matrix and each of the feature-based models and CNN-based models (Nili et al., 2014). This was performed using the Matlab function *partialcorr*, allowing us to quantify the unique contribution of each model, while controlling for correlations between models. In order to evaluate the impact of RMS contrast on both low-level and high-level category processing, the feature-based RDM partial correlation analysis was repeated with the RMS contrast-based RDM partialled out. For the purposes of this analysis, RMS contrast was defined as the standard deviation of pixel intensity values divided by mean intensity across each image (Scholte et al., 2009), and the contrast-based RDM consisted of pairwise Euclidean distances between stimulus RMS contrast values.

The significance of the coefficients was assessed through permutation testing, by shuffling the stimulus labels and recomputing the partial correlations 100 times for each ROI and time window. We used a one-sided test, as negative correlations between distance matrices were not expected and would be difficult to interpret (Furl et al., 2017). P-values obtained were thresholded using the maximum correlation coefficient across time points and the alpha was set to 0.01 to account for the number of models tested. This method only highlighted correlations that were stronger than all those in the empirical null distribution.

To assess the maximum possible correlation given the noise in the data, we used guidelines suggested by Nili et al. (2014). We computed an upper bound of the noise ceiling by correlating the average neural RDM across subjects to each individual's neural RDM for each ROI and time window (overfitting and thus overestimating the true model correlation), and a lower bound by correlating each individual's RDM to the average of the remaining 18 subjects' RDMs (underfitting and thus underestimating the correlation).

### Eye gaze data collection and analysis

An iViewX MEG250 eyetracker system (SensoMotoric Instruments) with a sampling rate of 250 Hz was used to track each subject's right pupil and corneal reflection during the MEG recordings. The camera was located in front of the participant at a distance of 120 cm. The system was calibrated using a 9-point calibration grid at the start of each session, and was recalibrated between sessions to account for changes in head position during the break.

Eye-tracker data was analyzed using EEGLAB (Delorme and Makeig, 2004), EYE-EEG (Dimigen et al., 2011), and Matlab R2015a. Vertical and horizontal eye gaze positions were recorded based on pupil position and were compared offline in order to assess differences between eye movement patterns across scene categories. After selecting time windows corresponding to the stimulus presentation (1 s post-stimulus onset), portions of missing eye-tracker data corresponding to blinks were reconstructed using linear interpolation prior to statistical analysis. Trials deviating from the mean by more than 2 standard deviations were excluded. We calculated the grand means, medians and standard deviations of eye gaze position for each condition and participant and tested for differences using two-way repeated measures ANOVAs with factors “Category” (levels “natural”, “urban”, and “scrambled”) and “Frequency” (levels “low”, “broadband”, and “high”). P-values were corrected for six comparisons (three tests on horizontal and vertical eye gaze data). No significant differences were found for either of the two factors (F (2,36)<2.57, *P*>0.09 (Category); F (2,36)<2.32, *P*>0.11 (Frequency); F (4,72)<2.55, *P*>0.04, alpha = 0.0083).

Next, we performed MVPA to test whether scene categories could be differentiated using single-trial eye gaze data. Gaze position values for the entire stimulus duration were entered as features in an initial analysis, while a subsequent analysis used time windows of 40 ms to check for time-resolved effects. Binary classification was performed on all six pairs of scene category conditions (scenes vs scrambled stimuli and natural vs urban scenes, for each spatial frequency condition). Accuracy did not exceed 51.98% (SD 6.08%) across participants for any of the 6 pairs of conditions tested. Time-resolved MVPA led to similar results (maximum accuracy over time and classification problems 53.69%, SD 5.94%).

## Results

### Behavioural categorization results

Participants were asked to categorize stimuli as scenes/scrambled and natural/urban respectively. Performance was high on both tasks (mean accuracy 95.27%, SD 5.63%, and 94.46%, SD 3.56% respectively; Supplementary Figure 3) and ranged between 90.47% and 98.45% across all conditions. We evaluated differences in performance and reaction time between spatial frequency conditions using one-way repeated ANOVAs.

Recognition performance did not significantly differ for scenes filtered at different spatial frequencies when participants had to make urban/natural judgements (F (1.78, 23.09) = 0.15, *P* = 0.83, *η*^2^ = 0.01). However, a significant difference was found when participants categorized stimuli as scenes or scrambled stimuli (F (1.47, 19.09) = 15.44, *P* = 0.0002, *η*^2^ = 0.54), with LSF images categorized significantly less accurately than broadband (t (13) = 3.08, *P* = 0.008, 95% CI [1.17, 24.43]) and HSF images (t (13) = 6.03, *P* = 4.24*10^−5^, 95% CI [9.48, 25.94]).

Responses were slightly slower on the scene vs scrambled task (mean raw RT 537 ms, SD 54 ms, versus 506 ms, SD 61 ms on the natural vs urban task). A one-way repeated measures ANOVA on mean log-transformed reaction times revealed a significant effect of frequency on the scene vs scrambled task (F (1.75,22.77) = 48.62, *P* = 1.4*10^−8^, *η*^2^ = 0.79), with Bonferroni-corrected follow-up tests revealing significantly slower reaction times for LSF images compared to both broadband images (t (13) = 8.37, *P* = 1.3*10^−6^, 95% CI [0.07, 0.15] and HSF images (t (13) = 6.92, *P* = 10^−5^, 95% CI [0.05, 0.12]). A smaller effect was found for the natural vs urban task (F (1.71, 22.25) = 6.11, *P* = 0.01, *η*^2^ = 0.32), with slower reaction times for LSF than HSF images revealed in follow-up tests (t (13) = 3.06, *P* = 0.009, 95% CI [0.01, 0.06]). Despite the effect reported here, we note that performance was above 90% on all conditions, suggesting high scene recognizability regardless of spatial frequency filtering.

### Decoding responses to unfiltered scene categories

#### Sensor-space decoding

**Fig. 4:**
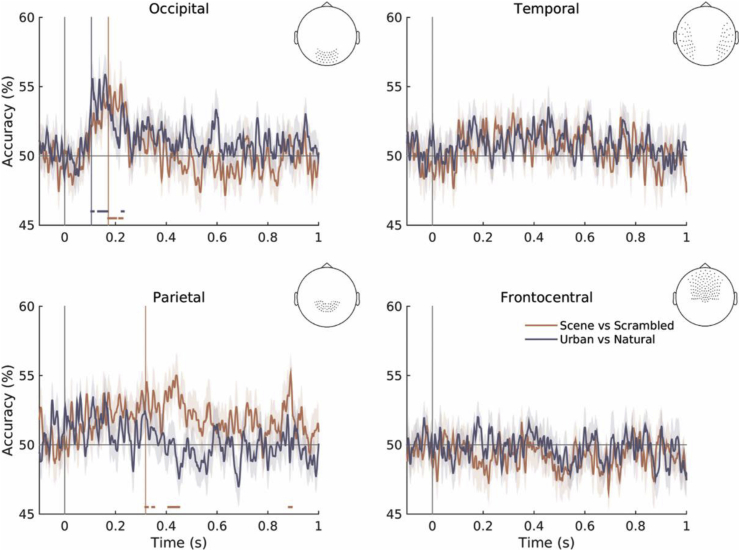
Time-resolved decoding accuracy traces (±SEM) obtained using different sensor sets for both decoding problems. Accuracies were averaged across subjects and smoothed with a five-point moving average for visualization only. Horizontal lines show above-chance decoding performance (*P* < 0.01 corrected).

To evaluate differences in neural responses between stimulus categories, we performed time-resolved decoding of responses to scenes vs scrambled stimuli and natural vs urban scenes using anatomically defined sensor sets. Above-chance decoding performance was achieved using the occipital sensor set starting at 172 ms and 105 ms post-stimulus onset respectively (Fig. 4). This effect was transient for both decoding problems; the return to chance level could reflect the absence of late task-related processing in our passive viewing paradigm. There was a significant difference between onset latencies for the two decoding problems (Z = 26.46, *P* < 0.001, 95% CI [13, 97] ms]), likely to reflect early decoding of systematic low-level differences between urban and natural stimuli (for example in terms of cardinal orientations). Classification on the parietal sensor set also achieved significance after 318 ms for the scene vs scrambled decoding problem, suggesting more sustained scene processing along the dorsal stream.

#### Source-space decoding

To spatially localize the effects revealed by sensor-space MVPA, we moved into source space and performed MVPA analysis of scene category processing using virtual source timecourses obtained through LCMV beamforming and an AAL atlas-based ROI searchlight approach.

**Fig. 5:**
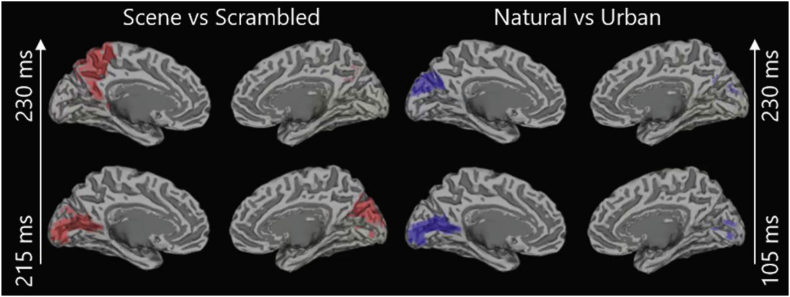
ROIs achieving significant decoding performance across subjects in the searchlight source-space MVPA analysis (*P* < 0.001, cluster-corrected across time).

Accuracies obtained in source space were comparable to sensor space performance (Supplementary Table 1). Early above-chance decoding was achieved for both problems in calcarine cortex (105 and 215 ms respectively) and along the dorsal stream for the scene versus scrambled decoding problem (∼230 ms; Fig. 5).

### From low-level to categorical representations

#### Within-frequency decoding

**Fig. 6:**
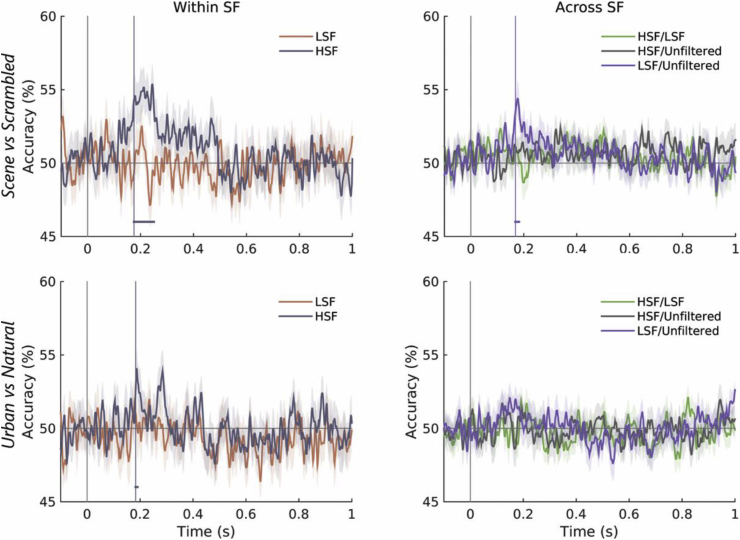
Time-resolved decoding accuracies (±SEM) for both decoding problems using the occipital sensor set. **Left:** decoding within spatial frequency (HSF and LSF); **Right:** cross-decoding across the broadband and LSF stimulus sets. Above-chance decoding time windows are marked with horizontal lines (*P* < 0.01 corrected).

To assess spatial frequency preferences in the processing of natural scenes, we performed within-spatial frequency and cross-spatial frequency classification using occipital sensor-level MEG responses. Only HSF stimuli achieved above-chance decoding performance in within-spatial frequency classification (Supplementary Table 2). Classification accuracy reached significance at 175 ms post-stimulus onset for the scene vs scrambled decoding problem, and briefly at 183 ms for the urban vs natural scene decoding problem (Fig. 6), thus following a similar timecourse to the decoding of unfiltered scenes.

#### Cross-frequency decoding

We performed cross-frequency decoding to evaluate the generalizability of scene responses across spatial frequencies. This allowed us to assess, for example, whether a decoder trained to classify scenes on a set of LSF stimuli could generalize to a set of HSF stimuli and vice versa.

We were unable to detect a truly high-level response (i.e., above-chance generalization across LSF and HSF stimulus sets). Successful cross-decoding was only achieved when classifying between scenes and scrambled stimuli across LSF and broadband stimulus sets (Fig. 6) starting at ∼168 ms after stimulus onset.

Contrast-related asymmetries in SNR pose a potential concern to this analysis (we note lower signal amplitudes in response to high spatial frequency, low contrast stimuli; see Supplementary Figure 5E). However, when decoding scenes from scrambled stimuli within each spatial frequency condition, higher accuracy was achieved on the HSF stimulus set than the higher contrast LSF set (Fig. 6), suggesting that discriminating information is present at high spatial frequencies despite lower SNR. The lower recognizability of LSF scenes (as shown in the behavioural experiment) may explain the lower accuracies obtained in their classification.

Despite this, cross-decoding results suggest that responses to unfiltered scenes are based on LSF features within 200 ms of stimulus onset. Successful cross-decoding points to a similarly structured multidimensional feature space across conditions, allowing successful generalization of the classifier decision boundary (Grootswagers et al., 2017). In our case, comparable results are achieved in both directions of training and testing, suggesting that despite lower classification rates within the LSF stimulus set, LSF features play an important role in natural scene perception. Although HSF features appear to contain information discriminating scenes from scrambled stimuli, it is more likely that these are associated with low-level perception, as they fail to generalize to broadband scene representations. Together, the MVPA analyses describe natural scene perception as a multi-stage process, with different spatial frequencies playing different roles in the encoding of information in visual cortex.

#### Low-level and categorical representations in visual cortex

**Fig. 7:**
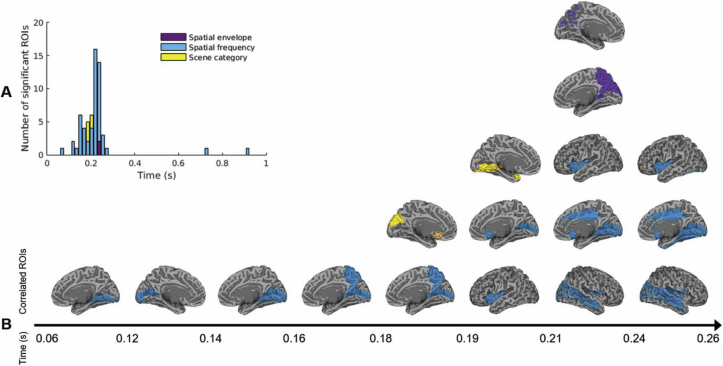
**A.** Number of ROIs significantly correlated with either of the feature-based models over time. **B.** Summary view of the ROIs significantly correlated with either of the feature-based models over time, overlaid on the MNI template brain (P < 0.01 corrected). For bilateral ROIs, one hemisphere is shown for clarity.

We interrogated the structure of neural representations using two RSA analyses. First, we performed RSA to test for partial correlations between MEG responses to scenes and four models guided by low-level properties or high-level category distinctions between stimuli. Neural patterns correlated most often and significantly with the spatial frequency-based model (maximum correlation *r* = 0.24, *P* < 0.01; Fig. 7A), with a few ROIs (shown below) showing significant correlations with the spatial envelope and scene category models (maximum *r* = 0.18 and *r* = 0.14 respectively, *P* < 0.01). No correlations with the scene identity model reached significance after correction for multiple comparisons (*r*<0.16, *P*>0.039).

The spatiotemporal evolution of different scene representations is shown in Fig. 7B. At early time points (before 150 ms), responses in early visual areas such as the lingual gyrus and calcarine cortex significantly correlated with the spatial frequency model, with correlations extending parietally and temporally later (150–250 ms). Interestingly, responses in posterior cingulate, temporal and extrastriate ROIs, where we might expect selective responses to scenes, correlated with the spatial frequency RDM at relatively late time points. These included areas identified in the MVPA analysis as supporting scene decoding.

Spatial envelope correlations were less represented in this dataset than reported by others (Ramkumar et al., 2016; Rice et al., 2014) and recruited occipito-parietal areas at ∼210 ms. Interestingly, these correlations appeared later than those with the scene category model, suggesting overlapping processing of low-, mid- and high-level properties in the visual system (Ramkumar et al., 2016).

While the scene identity model did not predict MEG patterns, the scene category model correlated with responses in the visual cortex at ∼180 ms post-stimulus onset. We note that correlations with the spatial frequency and the spatial envelope RDMs were partialled out of this analysis; it is thus likely that these correlations reflect true categorical differences in perception. This stage in processing coincides with the emergence of an occipital LSF scene response in the cross-decoding analysis (Fig. 6).

After excluding the contribution of the RMS contrast-based RDM from the partial correlation analysis, the spatial frequency sensitivity revealed earlier was diminished. This is in line with previous reports suggesting that spatial frequency processing is dependent on the amplitude spectrum (Andrews et al., 2010; Kauffmann et al., 2015a). RMS contrast also appeared to impact spatial envelope correlations, which arose later in this analysis (Fig. 10). Interestingly, significant correlations with the category-based model occurred at the same timepoints and in the same ROIs as in the previous analysis, reinforcing the idea that this is a truly high-level response.

**Fig. 8:**
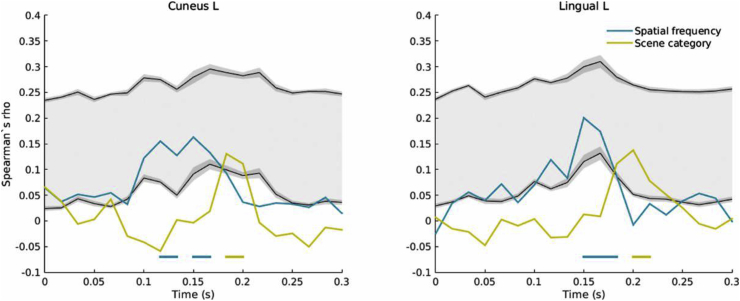
Example of correlation time-course (in steps of ∼16 ms) for the two visual cortex ROIs showing category-related representations. The gray shaded areas represent the noise ceiling, delineated by upper and lower bounds in black. The upper bound was calculated by correlating the average neural RDM across subjects to each individual's neural RDM, while the lower bound was obtained by correlating each individual's RDM to the average of the remaining 18 subjects' RDMs. 95% confidence intervals on the noise ceiling bounds are represented in dark gray. The horizontal lines show significant correlations arising when the correlation coefficient overlaps with the noise estimate, as expected (*P* < 0.01 corrected).

**Fig. 9:**
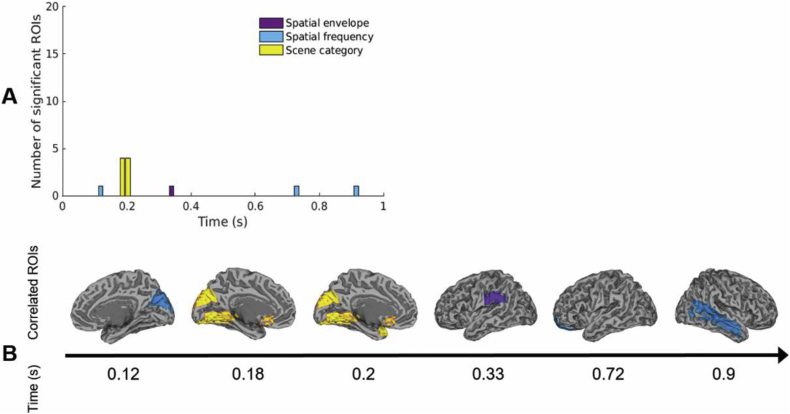
**A.** Number of ROIs significantly correlated with either of the feature-based models over time after partialling out the RMS contrast based model. **B.** Summary view of the ROIs significantly correlated with either of the feature-based models over time, overlaid on the MNI template brain (*P* < 0.01 corrected). Note that scene category correlations remain virtually unchanged.

**Fig. 10:**
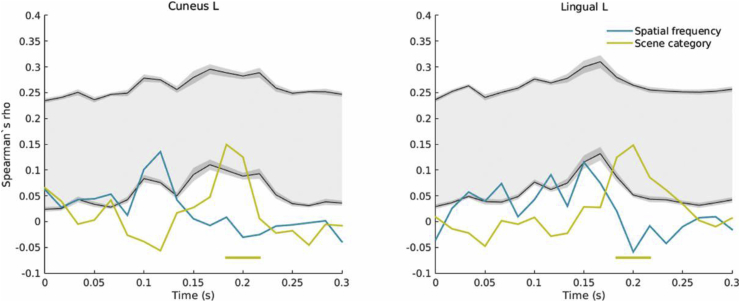
Example of correlation time-course for the two ROIs after partialling out RMS contrast.

While the correlation coefficients are relatively low, with a maximum of 5.7% of the variance explained by the spatial frequency model, the noise estimate suggests that the maximum correlation detectable in our data is low (mean lower and upper bound estimates across time and ROIs of *r* = 0.038 and *r* = 0.25 respectively; see Fig. 8, Fig. 10 for examples of time-resolved correlations compared to the noise ceiling). These values are comparable with previous RSA results obtained with similar data (Cichy et al., 2016a; Wardle et al., 2016), but higher SNR data (e.g. larger trial numbers) would be desirable to increase sensitivity (Nili et al., 2014).

#### Overlapping representations of CNN-based models

We performed a second analysis using model RDMs based on layers of a feedforward deep neural network to assess the hierarchy of scene representations in the visual system. Unsurprisingly given the high correlations between layer-specific RDMs (Fig. 3), only three layers achieved sustained significant partial correlations with the neural patterns: the second convolutional layer (starting at ∼80 ms), the first convolutional layer (starting at ∼150 ms), and the seventh fully connected layer (∼180–200 ms).

**Fig. 11:**
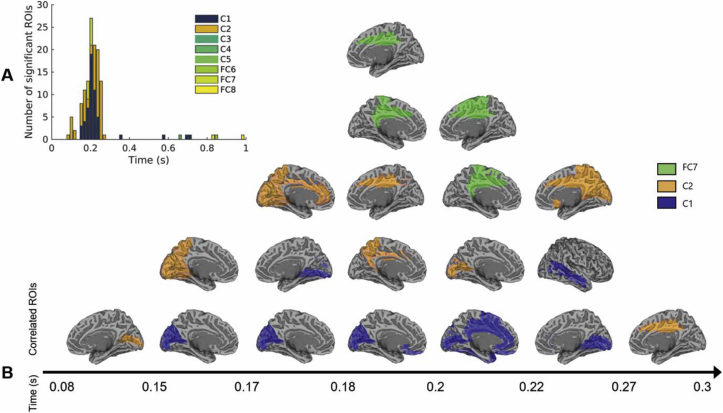
**A.** Number of ROIs significantly correlated with either of the CNN-based models over time. **B.** Summary view of the ROIs significantly correlated with either of the CNN-based models over time.

**Fig. 12:**
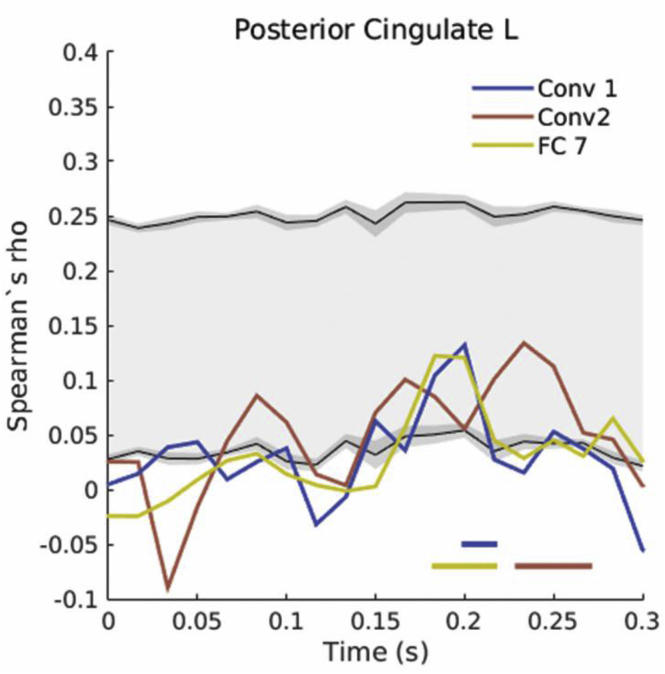
Time-course of correlations with CNN-based models in left posterior cingulate cortex.

In line with the results reported above, these representations were temporally and spatially overlapping both in visual cortex and higher-level cortices (Fig. 11). Interestingly, the high-level layer RDM was represented at the same time points as the categorical representations discussed above, but in higher-level areas including the posterior cingulate cortex. This highlights the potential of deep neural networks as a model that can explain representations in scene-selective cortex (as shown by recent fMRI work linking OPA patterns with CNN features: Bonner and Epstein, 2018); however, we note that at ∼180–200 ms, both the low-level and high-level CNN layers make significant unique contributions to explaining the variance in these ROIs (Fig. 12). Note also that the high-level CNN RDM is correlated to the low-level feature models (Fig. 3) and is more dependent on stimulus visual properties than the categorical models tested in the previous analysis. Thus, CNN-based representations paint a complementary picture to the feature-based models, while providing additional evidence against a low-to-high hierarchy of scene processing in the visual system.

## Discussion

Using natural and urban scene stimuli filtered at different spatial frequencies, we tracked the spatiotemporal dynamics of scene perception and tested for low-level and high-level representations of scenes using MEG. We report three main findings based on our analyses.

First, we used MVPA to reveal early (∼100 ms) scene processing in the visual cortex. Brain areas along the dorsal and ventral streams encoded information discriminating scenes from scrambled stimuli, while scene category was decodable mainly in visuoparietal cortex.

Second, we used a cross-decoding procedure with independent training and test sets to show the emergence of a response to scenes encoded at low spatial frequencies within 200 ms post-stimulus onset.

Finally, time-resolved RSA results revealed a high-level representation of scene category arising in extrastriate visual cortex at ∼180 ms. Both low-level and high-level brain areas contained spatial frequency representations, although these were shown to be dependent on RMS contrast. Furthermore, representations based on layers of a feedforward neural network correlated with visual system and higher-level regions in a temporally overlapping fashion, adding to the evidence of non-hierarchical processing of natural scenes.

### Temporal dynamics of scene processing

To date, there has not been extensive electrophysiological research into the temporal dynamics of natural scene processing. Previous studies have isolated responses to scenes by contrasting different types of scenes (Bastin et al., 2013; Cichy et al., 2016a; Groen et al., 2016, 2013), or scenes and faces (Rivolta et al., 2012; Sato et al., 1999) or objects (Harel et al., 2016); however, to our knowledge, no previous M/EEG study has used matched control stimuli, which are common in the fMRI literature on natural scenes.

While an early scene-specific event-related field (ERF) component has been reported (M100p: Rivolta et al., 2012), other studies only report late effects (after 200 ms; Groen et al., 2016; Harel et al., 2016; Sato et al., 1999). An MVPA study of natural scenes identified an early low-level response (100 ms) as well as a later signal associated with spatial layout (250 ms; Cichy et al., 2016a). In the current study, we report evidence of multiple stages in scene processing.

Although no early ERF differences are present in this dataset (possibly due to the matched control stimuli used; Supplementary Figure 5), the MVPA approach revealed single-trial differences starting at ∼100 ms for natural vs urban scenes, and at ∼170 ms for scenes vs scrambled stimuli. Classification of natural and urban scenes rose above chance significantly earlier than scene vs scrambled decoding; the occipital origin of this effect suggests a potential contribution of low-level systematic differences between stimulus categories. Successful cross-decoding occurred at similar time points and appeared to reflect a response to scenes based on LSF features, which may be reflected in the simultaneous significant correlations of neural patterns with a scene category model (Fig. 9). Information about scene category appeared to also be encoded in HSF features at the same time, although this did not generalize across stimulus categories. This response may thus reflect low-level differences encoded at high frequencies and is in line with previous studies showing evidence of responses to HSF images in scene-selective cortex (Berman et al., 2017). Together, these results point to divergent processing of features encoded at different spatial frequencies.

Interestingly, only the extrastriate visual cortex and an area in orbitofrontal cortex showed correlations with categorical scene representations, while the right temporal lobe contained persistent representations of spatial frequency and contrast (Fig. 7, Fig. 9). This suggests that visual features may play a part in driving responses in scene-selective areas. This is also supported by overlapping representations of low-level and high-level CNN layer models in areas such as posterior cingulate cortex. On the other hand, categorical responses beyond these areas may be differently represented or may be dependent on behavioural categorization goals.

### Mapping scene-selective responses

Extensive fMRI research has mapped responses to natural scenes to the visual cortex, OPA, PPA and RSC (e.g. Nasr et al., 2011; Walther et al., 2009). Here, we used MEG source-space MVPA to detect brain regions responding differently to scenes and scrambled stimuli, or natural and urban scenes respectively. We found differentiating information in visual and parietal cortex when decoding scenes and scrambled stimuli, with more focal patterns discriminating between natural and urban scenes. While the lower sensitivity of MEG to deep sources makes it challenging to detect responses in areas like the PPA, the sources reported here are in line with previous research reporting occipito-parietal sources of electrophysiological scene-responsive components (Groen et al., 2016; Rivolta et al., 2012).

Furthermore, the RSA mapping of correlations between neural responses and models based on low-level properties or categorical representations showed no classic low-to-high-level dissociation in the visual system. For example, spatial envelope correlations were strongest in occipito-parietal cortex at approximately 230 ms post-stimulus onset, similarly to previously reported correlations with MEG data (Ramkumar et al., 2016), and occurred later than categorical representations. Although not an exhaustive descriptor of scene properties, the spatial envelope model was chosen due to strong evidence that the GIST descriptor accurately represents global scene properties including naturalness, openness, and texture, which match representations in the human visual system (Oliva et al., 2001; Rice et al., 2014; Watson et al., 2017). Significant correlations in parietal areas suggest that scene-specific dorsal stream areas highlighted in the MVPA analysis may rely on image statistics. Finally, neural network representations explained posterior cingulate responses in a temporally and spatially overlapping manner, reinforcing the idea of a complex relationship between visual features and categorical representations.

#### Spatial frequency and RMS contrast

When contrast was not removed from the RSA analysis, spatial frequency-related representations appeared early (within 100 ms) in the primary visual cortex and extended along the dorsal stream (∼160 ms) and later along the ventral stream, as well as parietal and cingulate areas (∼200 ms). Despite the limited spatial resolution of MEG and of our ROI-based analysis, we note that correlations were strong in parahippocampal, parietal, cingulate, and inferior occipital areas corresponding to the reported locations of the PPA, RSC and OPA (Fig. 7). However, when we controlled for RMS contrast, spatial frequency representations only remained strong in visual cortex (∼120 ms) and, later, in high-level areas (orbitofrontal and temporal areas; Fig. 9). This is in line with previous reports showing spatial frequency processing in scene-selective areas (e.g. Nasr et al., 2014; Watson et al., 2016, 2014), as well as studies suggesting that such effects are dependent on the frequency-specific amplitude spectrum characteristic of natural scenes (Kauffmann et al., 2015b).

Spatial frequency has been previously shown to have a stronger effect on scene recognition than independent contrast manipulation, with low-frequency features leading to faster recognition; however, the interaction between RMS contrast and spatial frequency elicits the strongest behavioural effects (Kauffmann et al., 2015a). The distribution of contrast across spatial frequency follows a neurobiologically and behaviourally relevant pattern (Andrews et al., 2010; Bex et al., 2009; Guyader et al., 2004), and was maintained in the present study so as to avoid introducing irregularities in the amplitude spectra that would modify natural visual processing strategies. Importantly, contrast did not vary across high-level stimulus categories and only correlated with spatial frequency, ensuring that representations revealed in the MVPA and RSA analyses are contrast-independent.

#### Categorical representations

In our RSA analysis, category-related representations appeared relatively late in visual cortex, and could be speculatively linked to feedback mechanisms (Peyrin et al., 2010). The proximity of the ROIs to the transverse occipital sulcus suggests the OPA as a potential source of categorical representations.

The emergence of categorical representations at ∼180–200 ms post-stimulus onset coincides with previous reports of reaction times in human categorization of natural scenes. Some studies of gist perception report reaction times of at least 250 ms (Rousselet et al., 2005), but studies involving rapid categorization of scenes as natural or man-made interestingly report median reaction times of approximately 200 ms (Crouzet et al., 2012; Joubert et al., 2007). Our data show that at approximately 180 ms the categorical model supersedes the spatial frequency model in visual cortex, while low-level features are simultaneously processed in higher-level areas (Fig. 7).

#### CNN layer representations

Previous research has highlighted the potential of CNNs as powerful models in explaining representations in object- and scene-selective cortex (Groen et al., 2018; Güçlü and van Gerven, 2014; Khaligh-Razavi and Kriegeskorte, 2014; Yamins and DiCarlo, 2016), while an improving understanding of the feature representations employed by CNNs may in turn shed light on the mechanisms underpinning this link (Bonner and Epstein, 2018). In the current study, we extracted layer-specific representations in order to evaluate whether cortical patterns follow the hierarchy of a CNN. We found that high-level CNN representations occurred at the same time as the categorical representations discussed above (and coincided with successful decoding performance in the MVPA analysis). CNN-based models correlated significantly with areas along the dorsal stream, as well as higher-level areas such as the cingulate cortex, with convolutional and fully-connected layers contributing unique information to explaining temporally and spatially overlapping cortical patterns.

It is important to note that in both MVPA and RSA analyses, lack of decodable information or significant correlations does not constitute definitive evidence, as information may be otherwise represented in the neural data. However, by comparing multiple models, we provide evidence of the evolution of neural representations in time and space. While the RSA analysis of neural network representations does not match a simple hierachical view of scene processing, it highlights CNN features as good candidate models in explaining scene-selective cortex representations, in line with previous research (Seeliger et al., 2017; Yamins et al., 2014). On the other hand, the feature-based RSA analysis sees categorical representations arise independently of spatial frequency, RMS contrast, spatial frequency and scene identity, which, unlike the spatial frequency/contrast-based representations (Fig. 7, Fig. 9), do not involve V1. While early differences in our MVPA analysis may be driven by local low-level differences between scene categories, the RSA analysis points to a later categorical response, simultaneous with the response to low spatial frequencies identified in our cross-decoding analysis.

### What's in a category?

A growing body of work suggests that low-level properties play an important part at all stages of processing in the emergence of category-specific representations (Groen et al., 2017). Thus, MVPA analysis results can be difficult to interpret. Even though the stimuli used in our experiment were normalized in terms of Fourier amplitudes and spatial frequency, a number of properties remain that may differentiate between any two categories, such as the number of edges or the spatial envelope. While it is to be expected that differences in visual properties underpin any differences in high-level representations, assessing the role of low-level properties can help elucidate the source of pattern differences found in our study. Thus, the cross-decoding and RSA analyses provide additional evidence of a categorical stage in natural scene perception and help differentiate this from the earlier, visually driven response revealed by MVPA.

The present study used a passive viewing paradigm, which approached natural viewing conditions and ensured that category effects were not driven by task-related processing, while still controlling for low-level confounds. In the absence of a categorization task, we failed to detect a truly high-level response in our cross-decoding analysis (i.e., generalization across low and high frequency stimuli; Fig. 6). However, the scene-specific response revealed in the decoding analysis generalized across unfiltered and low spatial frequency stimuli within 200 ms, suggesting that low frequency cues encode scene-specific information at later stages of scene processing. Future studies could apply a cross-decoding procedure to data collected using a categorization task in order to investigate the presence of a frequency-invariant response.

Furthermore, we note that failure to achieve above-chance decoding performance in LSF decoding or cross-decoding does not preclude the existence of differential responses that are otherwise represented in the brain, or that the current study design did not detect. However, the current results are informative in comparing conditions and linking the decodability of stimulus categories to spatial frequency information, thus pointing to preferences in spatial frequency processing that may underpin the rapid perception of natural scenes.

Although the repetition of a limited set of stimuli across different spatial frequencies has advantages in terms of controlling for low-level properties, this also poses the concern of stimuli being recognizable between spatial frequency conditions, thus potentially affecting the category differences observed here. However, the fact that we were unable to cross-decode LSF and HSF scenes suggests that such a recognition response could not have significantly contributed to decoding results. Furthermore, such recognition would be expected to affect all conditions equally (given the stimulus randomization procedure), and would therefore not explain the spatial frequency-specific effects reported here. Finally, we included a scene identity model RDM in our feature-based RSA analysis to assess the recognition of individual scenes across spatial frequency conditions and found no significant correlations with the neural patterns. However, future studies could alleviate this concern by including a larger number of stimuli.

Scene perception is understood as involving a coarse-to-fine processing sequence using both low spatial frequency cues (rapidly processed and allowing for parsing of global structure) and high frequency information (which is relayed more slowly to high-level areas; Kauffmann et al., 2014). The present study links the rapid processing of low frequency cues to the formation of categorical representations, supporting previous reports of coarse visual analysis as rapid and crucial to gist perception (Kauffmann et al., 2017; Peyrin et al., 2010; Schyns and Oliva, 1994). On the other hand, high spatial frequency representations of scenes do not generalize to unfiltered stimuli, suggesting that they may encode low-level differences rather than a categorical response. However, the presence of such a response may reflect HSF representations previously found in visual and scene-selective areas (Berman et al., 2017; Walther et al., 2011).

Behavioural results obtained through a separate experiment revealed that scenes filtered at low spatial frequencies are more difficult to distinguish from scrambled stimuli than unfiltered or highpass-filtered scenes. This difference was reflected in the lower decodability of LSF scenes from scrambled stimuli. Low-frequency scenes thus appear to be more similar to their scrambled counterparts; interestingly, the similarity in contrast between low-frequency and unfiltered scenes does not provide a categorization or decoding advantage.

However, the difference between the categorization task in the behavioural experiment, with its speed/accuracy tradeoff, and the passive viewing paradigm used in the MEG, means that behavioural results need to be interpreted cautiously. The high behavioural performance across participants (over 90%) suggests that despite these differences, stimuli were generally recognizable across categories.

Challenging traditional ideas of a low-to-high-level hierarchy in the visual system, recent studies have emphasized the role of low-level properties in scene-selective perception, while at the same time suggesting that categorical distinctions play an important role in behavioural decision-making (Rice et al., 2014; Watson et al., 2016). Such distinctions may emerge from image features and are not “explained away” by low-level properties (Groen et al., 2017; Watson et al., 2017). Here, we take a step further in explaining how high-level representations arise from the processing of visual features. The RSA and cross-decoding results suggest that spatial frequency is relevant in scene perception, with low-frequency features carrying the information identifying natural scenes as such. Within 200 ms, the human visual cortex switches from a low-level representation of stimuli to a categorical representation independent of spatial frequency, contrast and spatial envelope. Furthermore, a convolutional neural network explains representations in visual and cingulate cortex, with high-level layers being represented within 200 ms. As these representations arise in the absence of a task, our results describe a visual system highly adapted to rapidly extracting information from the environment, an important asset in navigating and understanding our everyday surroundings.

## Appendix A Supplementary data

DimaDC_SupplementaryMaterial DimaDC_SupplementaryMaterial

## References

[bib1] Andrews T.J., Clarke A., Pell P., Hartley T. (2010). Selectivity for low-level features of objects in the human ventral stream. Neuroimage.

[bib2] Bastin J., Committeri G., Kahane P., Galati G., Minotti L., Lachaux J.P., Berthoz A. (2013). Timing of posterior parahippocampal gyrus activity reveals multiple scene processing stages. Hum. Brain Mapp..

[bib3] Berman D., Golomb J.D., Walther D.B. (2017). Scene content is predominantly conveyed by high spatial frequencies in scene-selective visual cortex. PLoS One.

[bib4] Bex P.J., Solomon S.G., Dakin S.C. (2009). Contrast sensitivity in natural scenes depends on edge as well as spatial frequency structure. J. Vis..

[bib5] Bonner M.F., Epstein R.A. (2018). Computational Mechanisms Underlying Cortical Responses to the Affordance Properties of Visual Scenes.

[bib86] Brainard D.H. (1997). The psychophysics toolbox. Spatial Vis..

[bib6] Cichy R.M., Khosla A., Pantazis D., Oliva A. (2016). Dynamics of scene representations in the human brain revealed by magnetoencephalography and deep neural networks. Neuroimage.

[bib7] Cichy R.M., Khosla A., Pantazis D., Torralba A. (2016). Comparison of deep neural networks to spatio-temporal cortical dynamics of human visual object recognition reveals hierarchical correspondence. Nat. Publ. Gr.

[bib8] Cohen J. (1992). A power primer. Psychol. Bull..

[bib9] Crouzet S.M., Joubert O.R., Thorpe S.J., Fabre-Thorpe M. (2012). Animal detection precedes access to scene category. PLoS One.

[bib10] Delorme A., Makeig S. (2004). EEGLAB: an open source toolbox for analysis of single-trial EEG dynamics including independent component analysis. J. Neurosci. Meth..

[bib11] Dimigen O., Sommer W., Hohlfeld A., Jacobs A.M., Kliegl R. (2011). Coregistration of eye movements and EEG in natural reading: analyses and review. J. Exp. Psychol. Gen..

[bib12] Etzel J.A., Zacks J.M., Braver T.S. (2013). Searchlight analysis: promise, pitfalls, and potential. Neuroimage.

[bib13] Felleman D.J., Van Essen D.C. (1991). Distributed hierachical processing in the primate cerebral cortex. Cerebr. Cortex.

[bib14] Field D.J. (1987). Relations between the statistics of natural images and the response properties of cortical cells. J. Opt. Soc. Am. A.

[bib15] Furl N., Lohse M., Pizzorni-Ferrarese F. (2017). Low-frequency oscillations employ a general coding of the spatio-temporal similarity of dynamic faces. Neuroimage.

[bib16] Groen I.I., Greene M.R., Baldassano C., Fei-Fei L., Beck D.M., Baker C.I. (2018). Distinct contributions of functional and deep neural network features to representational similarity of scenes in human brain and behavior. Elife.

[bib17] Groen I.I.A., Ghebreab S., Lamme V.A.F., Scholte H.S. (2016). The time course of natural scene perception with reduced attention. J. Neurophysiol..

[bib18] Groen I.I.A., Ghebreab S., Prins H., Lamme V.A.F., Scholte H.S. (2013). From image statistics to scene gist: evoked neural activity reveals transition from low-level natural image structure to scene category. J. Neurosci..

[bib19] Groen I.I.A., Silson E.H., Baker C.I. (2017). Contributions of low- and high-level properties to neural processing of visual scenes in the human brain. Philos. Trans. R. Soc. London. Ser. B, Biol. Sci..

[bib20] Grootswagers T., Wardle S.G., Carlson T.A. (2017). Decoding dynamic brain patterns from evoked responses: a tutorial on multivariate pattern analysis applied to time-series neuroimaging data. J. Cognit. Neurosci..

[bib21] Güçlü U., van Gerven M.A.J. (2014).

[bib22] Guyader N., Chauvin A., Peyrin C., Hérault J., Marendaz C. (2004). Image phase or amplitude? Rapid scene categorization is an amplitude-based process. Comptes Rendus Biol..

[bib23] Harel A., Groen I.I.A., Kravitz D.J., Deouell L.Y., Baker C.I. (2016). The temporal dynamics of scene processing: a multifaceted EEG investigation. eNeuro.

[bib24] Hillebrand A., Barnes G.R., Bosboom J.L., Berendse H.W., Stam C.J. (2012). Frequency-dependent functional connectivity within resting-state networks : an atlas-based MEG beamformer solution. Neuroimage.

[bib25] Hillebrand A., Singh K.D., Holliday I.E., Furlong P.L., Barnes G.R. (2005). A new approach to neuroimaging with magnetoencephalography. Hum. Brain Mapp..

[bib26] Hubel D.H., Wiesel T.N. (1962). Receptive fields, binocular interaction and functional architecture in the cat's visual cortex. J. Physiol..

[bib27] Jamalabadi H., Alizadeh S., Sch M., Leibold C., Gais S. (2016). Classification based hypothesis testing in Neuroscience : below-chance level classification rates and overlooked statistical properties of linear parametric classifiers. Hum. Brain Mapp..

[bib28] Jas M., Engemann D.A., Bekhti Y., Raimondo F., Gramfort A. (2017). Autoreject: automated artifact rejection for MEG and EEG data. Neuroimage.

[bib29] Jia Y., Shelhamer E., Donahue J., Karayev S., Long J., Girshick R., Guadarrama S., Darrell T. (2014). Caffe: convolutional architecture for fast feature embedding. Proc. ACM Int. Conf. Multimed. - MM ’14.

[bib30] Joubert O.R., Rousselet G.A., Fize D., Fabre-Thorpe M. (2007). Processing scene context: fast categorization and object interference. Vis. Res..

[bib31] Kauffmann L., Chauvin A., Guyader N., Peyrin C. (2015). Rapid scene categorization: role of spatial frequency order, accumulation mode and luminance contrast. Vis. Res..

[bib32] Kauffmann L., Ramanoël S., Guyader N., Chauvin A., Peyrin C. (2015). Spatial frequency processing in scene-selective cortical regions. Neuroimage.

[bib33] Kauffmann L., Ramanoël S., Peyrin C. (2014). The neural bases of spatial frequency processing during scene perception. Front. Integr. Neuroscience.

[bib34] Kauffmann L., Roux-Sibilon A., Beffara B., Mermillod M., Guyader N., Peyrin C., Kauffmann L., Roux-sibilon A., Beffara B. (2017). How does information from low and high spatial frequencies interact during scene categorization. Vis. cogn.

[bib35] Khaligh-Razavi S.M., Kriegeskorte N. (2014). Deep supervised, but not unsupervised, models may explain it cortical representation. PLoS Comput. Biol..

[bib36] Kinsey K., Anderson S.J., Hadjipapas a, Holliday I.E. (2011). The role of oscillatory brain activity in object processing and figure-ground segmentation in human vision. Int. J. Psychophysiol..

[bib87] Kleiner M., Brainard D.H., Pelli D.G., Broussard C., Wolf T., Niehorster D. (2007). What’s new in Psychtoolbox-3?. Perception.

[bib37] Kravitz D.J., Peng C.S., Baker C.I. (2011). Real-World scene representations in high-level visual cortex: it's the spaces more than the places. J. Neurosci..

[bib38] Kriegeskorte N., Goebel R., Bandettini P. (2006). Information-based functional brain mapping. Proc. Natl. Acad. Sci. Unit. States Am..

[bib39] Kriegeskorte N., Kievit R.A. (2013). Representational geometry : integrating cognition, computation, and the brain. Trends Cognit. Sci..

[bib40] Kriegeskorte N., Mur M., Bandettini P. (2008). Representational similarity analysis - connecting the branches of systems neuroscience. Front. Syst. Neurosci..

[bib41] Krizhevsky A., Sutskever I., Hinton G.E. (2012). ImageNet classification with deep convolutional neural networks. Adv. Neural Inf. Process. Syst..

[bib42] Lakens D. (2017). Equivalence tests: a practical primer for t tests, correlations, and meta-analyses. Soc. Psychol. Personal. Sci..

[bib43] Nasr S., Echavarria C.E., Tootell R.B.H. (2014). Thinking outside the box: rectilinear shapes selectively activate scene-selective cortex. J. Neurosci..

[bib44] Nasr S., Liu N., Devaney K.J., Yue X., Rajimehr R., Ungerleider L.G., Tootell R.B.H. (2011). Scene-selective cortical regions in human and nonhuman primates. J. Neurosci..

[bib45] Nasr S., Tootell R.B.H. (2012). A cardinal orientation bias in scene-selective visual cortex. J. Neurosci..

[bib46] Nichols T.E., Holmes A.P. (2001). Nonparametric permutation tests for functional Neuroimaging : a primer with examples. Hum. Brain Mapp..

[bib47] Nili H., Wingfield C., Walther A., Su L., Marslen-Wilson W., Kriegeskorte N. (2014). A toolbox for representational similarity analysis. PLoS Comput. Biol..

[bib48] Nilsson R., Pena J.M., Bjorkegren J., Tegner J. (2006). Evaluating feature selection for SVMs in high dimensions. Lect. Notes Comput. Sci..

[bib49] Noirhomme Q., Lesenfants D., Gomez F., Soddu A., Schrouff J., Garraux G., Luxen A., Phillips C., Laureys S. (2014). Biased binomial assessment of cross-validated estimation of classification accuracies illustrated in diagnosis predictions. NeuroImage Clin.

[bib50] Nolan H., Whelan R., Reilly R.B. (2010). FASTER : fully automated statistical thresholding for EEG artifact rejection ଝ. J. Neurosci. Meth..

[bib51] Norman K.A., Polyn S.M., Detre G.J., Haxby J.V. (2006).

[bib52] Oliva A., Hospital W., Ave L. (2001). Modeling the shape of the Scene : a holistic representation of the spatial envelope. Int. J. Comput. Vis..

[bib88] Oostenveld R., Fries P., Maris E., Schoffelen J.M. (2011). FieldTrip: open source software for advanced analysis of MEG, EEG, and invasive electrophysiological data. Comput. Intell. Neurosci..

[bib89] Pelli D.G. (1997). The VideoToolbox software for visual psychophysics: transforming numbers into movies. Spatial Vis..

[bib53] Perry G., Singh K.D. (2014). Localizing evoked and induced responses to faces using magnetoencephalography. Eur. J. Neurosci..

[bib54] Peyrin C., Michel C.M., Schwartz S., Thut G., Seghier M., Landis T., Marendaz C., Vuilleumier P. (2010). The neural substrates and timing of top-down processes during coarse-to-fine categorization of visual scenes: a combined fMRI and ERP study. J. Cognit. Neurosci..

[bib55] Rajaei K., Mohsenzadeh Y., Ebrahimpour R., Khaligh-Razavi S.-M. (2018). Beyond Core Object Recognition: Recurrent Processes Account for Object Recognition under Occlusion.

[bib56] Rajimehr R., Devaney K.J., Bilenko N.Y., Young J.C., Tootell R.B.H. (2011). The “parahippocampal place area” responds preferentially to high spatial frequencies in humans and monkeys. PLoS Biol..

[bib57] Ramkumar P., Hansen B.C., Pannasch S., Loschky L.C. (2016). Visual information representation and rapid scene categorization are simultaneous across cortex: an MEG study. Neuroimage.

[bib58] Rice G.E., Watson D.M., Hartley T., Andrews T.J. (2014). Low-level image properties of visual objects predict patterns of neural response across category-selective regions of the ventral visual pathway. J. Neurosci..

[bib59] Ritchie J.B., Carlson T.A. (2016). Neural decoding and “inner” Psychophysics: a distance-to-bound approach for linking mind, brain, and behavior. Front. Neurosci..

[bib60] Rivolta D., Palermo R., Schmalzl L., Williams M. a (2012). An early category-specific neural response for the perception of both places and faces. Cognit. Neurosci..

[bib61] Rousselet G.A., Joubert O.R., Fabre-thorpe Á. (2005). How long to get to the `` gist ’ ’ of real-world natural scenes. Vis. cogn.

[bib62] Sato N., Nakamura K., Nakamura a, Sugiura M., Ito K., Fukuda H., Kawashima R. (1999). Different time course between scene processing and face processing: a MEG study. Neuroreport.

[bib63] Schindler A., Bartels A. (2016). Visual high-level regions respond to high-level stimulus content in the absence of low-level confounds. Neuroimage.

[bib64] Scholte H.S., Smeulders A.W.M., Lamme V.A.F. (2009). Brain responses strongly correlate with Weibull image statistics when processing natural images. J. Vis..

[bib65] Schyns P.G., Oliva A. (1994). Evidence for time- and spatial-scale-dependent scene recognition. Psychol. Sci..

[bib66] Seeliger K., Fritsche M., Güçlü U., Schoenmakers S., Schoffelen J., Bosch S.E. (2017). NeuroImage Convolutional neural network-based encoding and decoding of visual object recognition in space and time. Neuroimage.

[bib67] Singh K.D., Barnes G.R., Hillebrand A. (2003).

[bib68] Smith S.M. (2002). Fast robust automated brain extraction. Hum. Brain Mapp..

[bib69] Studebaker G.A. (1985). A “rationalized” arcsine transform. J. Speech Hear. Res..

[bib70] Tzourio-Mazoyer N., Landeau B., Papathanassiou D., Crivello F., Etard O., Delcroix N., Mazoyer B., Joliot M. (2002). Automated anatomical labeling of activations in SPM using a macroscopic anatomical parcellation of the MNI MRI single-subject brain. Neuroimage.

[bib71] Ungerleider L.G., Haxby J.V. (1994). “What” and “where” in the human brain. Curr. Opin. Neurobiol..

[bib72] Van Veen B., van Drongelen W., Yuchtman M., Suzuki A. (1997). Localization of brain electrical activity via linearly constrained minimum variance spatial filtering. IEEE Trans. Biomed. Eng..

[bib73] VanRullen R., Thorpe S.J. (2001). The time course of visual processing: from early perception to decision-making. J. Cognit. Neurosci..

[bib74] Vrba J., Robinson S.E. (2001). Signal processing in magnetoencephalography. Methods.

[bib75] Walther D.B., Caddigan E., Fei-Fei L., Beck D.M. (2009). Natural scene categories revealed in distributed patterns of activity in the human brain. J. Neurosci..

[bib76] Walther D.B., Chai B., Caddigan E., Beck D.M., Fei-Fei L. (2011). Simple line drawings suffice for functional MRI decoding of natural scene categories. Proc. Natl. Acad. Sci. U. S. A.

[bib77] Wardle S.G., Kriegeskorte N., Grootswagers T., Khaligh-razavi S., Carlson T.A. (2016). Perceptual similarity of visual patterns predicts dynamic neural activation patterns measured with MEG. Neuroimage.

[bib78] Watson D.M., Andrews T.J., Hartley T. (2017). A data driven approach to understanding the organization of high-level visual cortex. Sci. Rep..

[bib79] Watson D.M., Hartley T., Andrews T.J. (2014). Patterns of response to visual scenes are linked to the low-level properties of the image. Neuroimage.

[bib80] Watson D.M., Hymers M., Hartley T., Andrews T.J. (2016). Patterns of neural response in scene-selective regions of the human brain are affected by low-level manipulations of spatial frequency. Neuroimage.

[bib81] Willenbockel V., Sadr J., Fiset D., Horne G.O., Gosselin F., Tanaka J.W. (2010). Controlling low-level image properties: the SHINE toolbox. Behav. Res. Meth..

[bib82] Xiao J., Hays J., Ehinger K.A., Torralba A. (2010). SUN Database : large-scale scene recognition from abbey to zoo. Comput. Vis. Pattern recognit. (CVPR), 2010 IEEE Conf.

[bib83] Yamins D.L.K., DiCarlo J.J. (2016). Using goal-driven deep learning models to understand sensory cortex. Nat. Neurosci..

[bib84] Yamins D.L.K., Hong H., Cadieu C.F., Solomon E.A., Seibert D., Dicarlo J.J. (2014). Performance-optimized Hierarchical Models Predict Neural Responses in Higher Visual Cortex.

[bib85] Zhou B., Lapedriza A., Xiao J., Torralba A., Oliva A. (2014). Learning deep features for scene recognition using places database. Adv. Neural Inf. Process. Syst..

