# Supplementary material for: Spatial frequency supports the emergence of categorical representations in visual cortex during natural scene perception
Source: Neuroimage. 2018 Oct 1;179:102–16. doi: 10.1016/j.neuroimage.2018.06.033 (PMC6057270; doi:10.1016/j.neuroimage.2018.06.033)
Supplement: DimaDC_SupplementaryMaterial [file mmc1.docx]

|  |  | Occipital | Temporal | Parietal | Frontocentral | Source space |
| --- | --- | --- | --- | --- | --- | --- |
| Scene vs Scrambled | Max accuracy | 56.07% | 53.93% | 56.12% | 52.93% | 57.41% |
|  | Bootstrap 95% CI | 53.21%, 60.59% | 51.14%, 57.4% | 53.24%, 59.21% | 50.79%, 55.78% | 54.4%,  60% |
|  | Max sensitivity | 58.95% | 56.9% | 58.38% | 54.73% | 69.8% |
|  | Max specificity | 54.96% | 53.58% | 55.54% | 53.34% | 57.07% |
|  | Decoding onset | 172 ms | N/A | 318 ms | N/A | 215 ms |
|  | Bootstrap 95% CI | 145-215 ms | N/A | 83-423 ms | N/A | 173-223 ms |
|  |  |  |  |  |  |  |
| Natural vs Urban | Max accuracy | 56.56% | 54.55% | 54.65% | 54.18% | 55.91% |
|  | Bootstrap 95% CI | 53.84%, 59.67% | 52.13%, 56.94% | 51.62%, 57.91% | 51.61%, 56.63% | 53.55%, 58.49% |
|  | Max sensitivity | 57.03% | 55.3% | 55.07% | 52% | 55.25% |
|  | Max specificity | 58.2% | 55.72% | 56.79% | 56.39% | 74.06% |
|  | Decoding onset | 105 ms | N/A | N/A | N/A | 105 ms |
|  | Bootstrap 95% CI | 102-146 ms | N/A | N/A | N/A | 102-232 ms |

**Supplementary Material**

**Supplementary Table 1.** Decoding performance based on different sensor sets and the source space searchlight approach.

|  |  | *Within Spatial Frequency* | | *Across Spatial Frequency* | | |
| --- | --- | --- | --- | --- | --- | --- |
|  | *Stimulus sets* | LSF | HSF | LSF-HSF | HSF-Unfilt | LSF-Unfilt |
| Scene vs Scrambled | Max accuracy | 54.63% | 56.43% | 53% | 53.42% | 55.3% |
|  | Bootstrap 95% CI | 52.24%, 58.73% | 54.41%, 58.23% | 51.51%, 55.7% | 51.96%, 54.75% | 53.65%, 57.94% |
|  | Max sensitivity | 54.47% | 55.85% | 53.76% | 56.66% | 56.22% |
|  | Max specificity | 56.95% | 57.31% | 54.97% | 54.57% | 54.97% |
|  | Decoding onset | N/A | 175 ms | N/A | N/A | 168 ms |
|  | Bootstrap 95% CI | N/A | 133-208 ms | N/A | N/A | 165-177 ms |
|  |  |  |  |  |  |  |
| Natural vs Urban | Max accuracy | 53.17% | 54.97% | 52.82% | 53.29% | 52.91% |
|  | Bootstrap 95% CI | 49.06%, 56.59% | 51.41%, 59.41% | 51.56%, 54.57% | 51.73%, 55.2% | 50.89%, 54.62% |
|  | Max sensitivity | 54.82% | 56.38% | 54.33% | 53.69% | 52.58% |
|  | Max specificity | 55.15% | 56.38% | 55.75% | 56.95% | 57.31% |
|  | Decoding onset | N/A | 183 ms | N/A | N/A | N/A |
|  | Bootstrap 95% CI | N/A | 183-282 | N/A | N/A | N/A |

**Supplementary Table 2.** Within-spatial frequency and cross- spatial frequency decoding results.


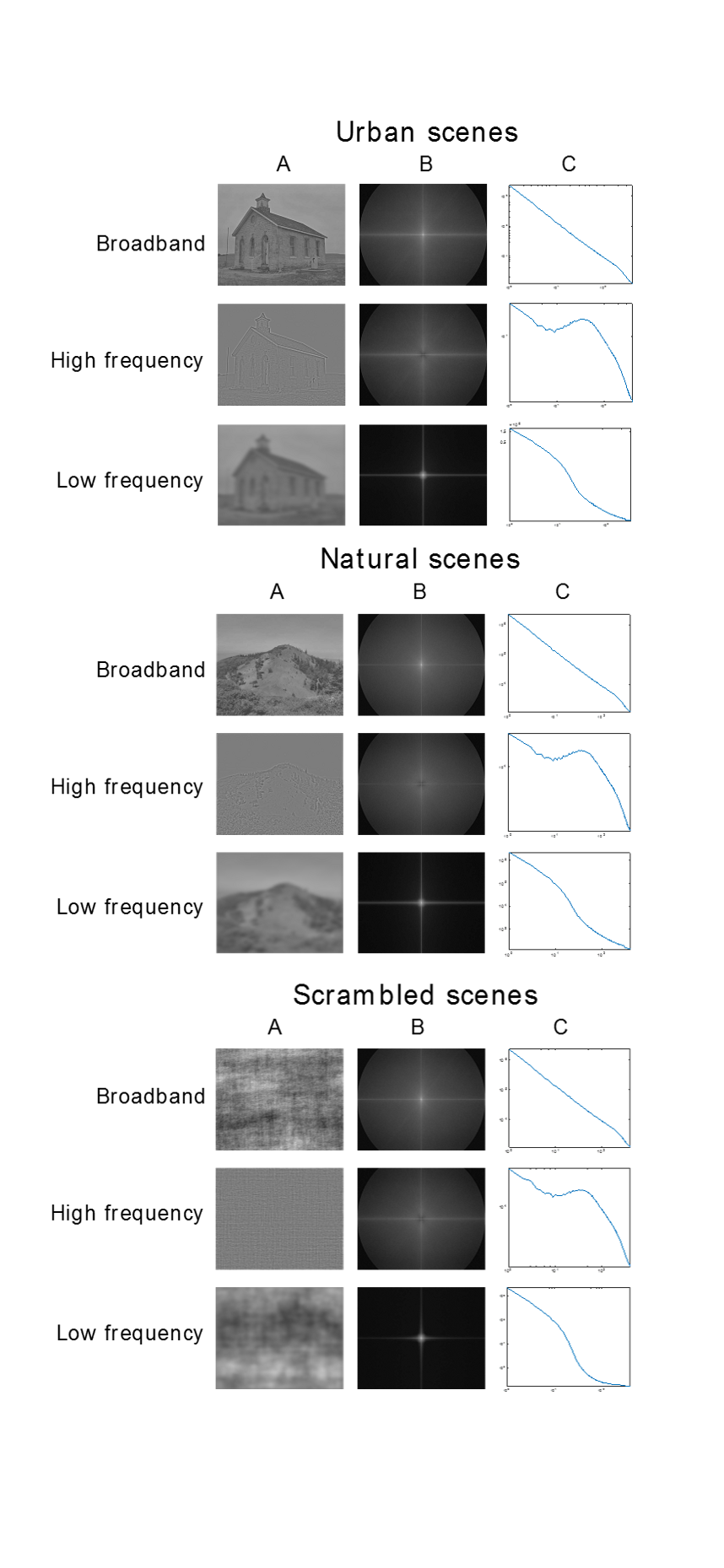


**Supplementary Figure 1.** Stimulus examples from all 9 conditions (A), together with average Fourier amplitude spectra (B) and spatial frequency plots (C) for each condition.


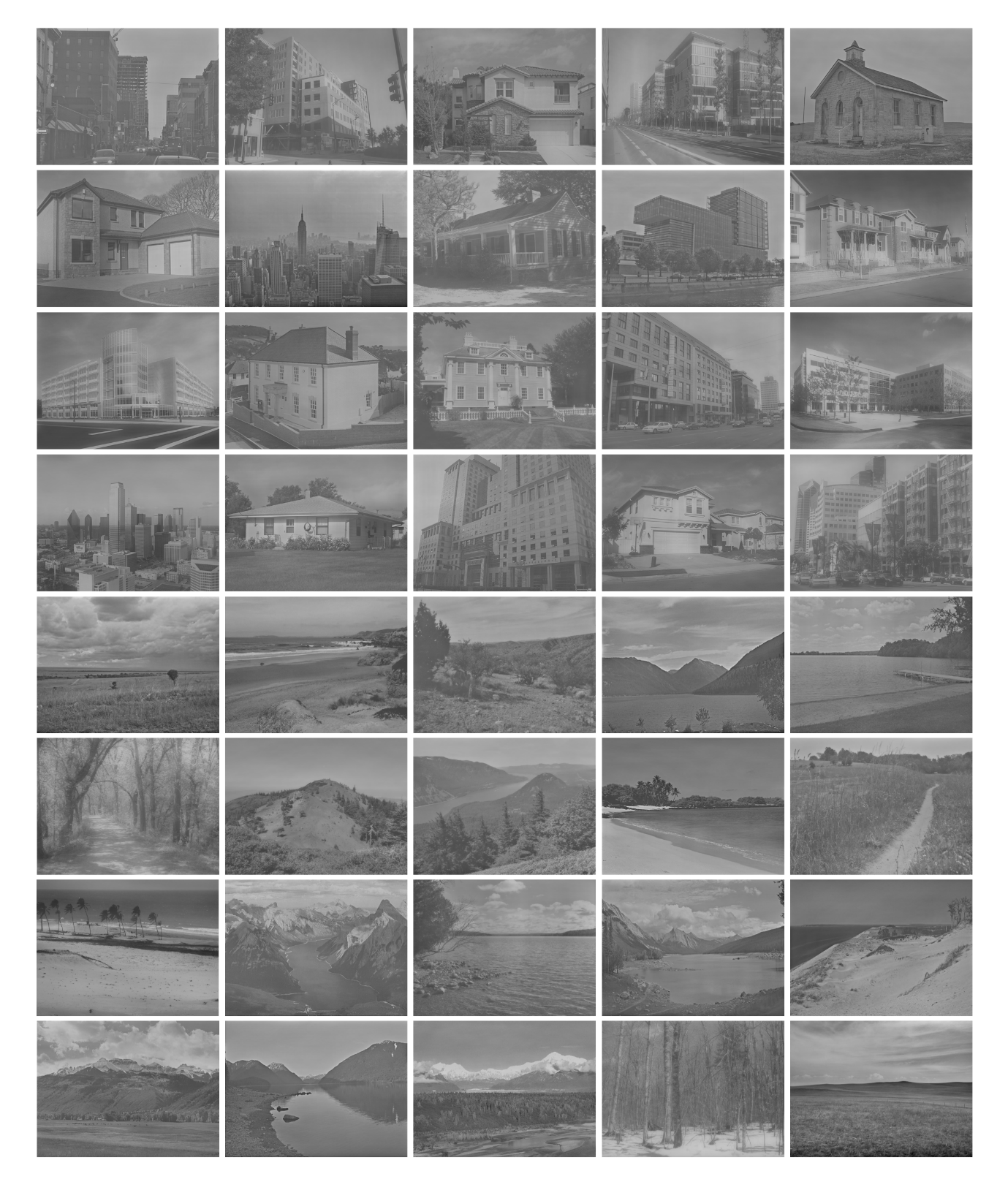


**Supplementary Figure 2.** The 40 scene stimuli used in the study (above: urban scenes; below: natural scenes).


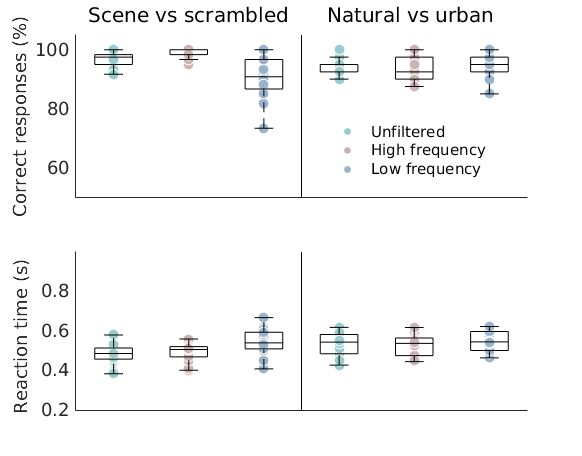


**Supplementary Figure 3.** Categorization performance and mean reaction times for the 14 participants in the behavioural experiment, represented separately for each of the spatial frequency conditions used in the experiment.


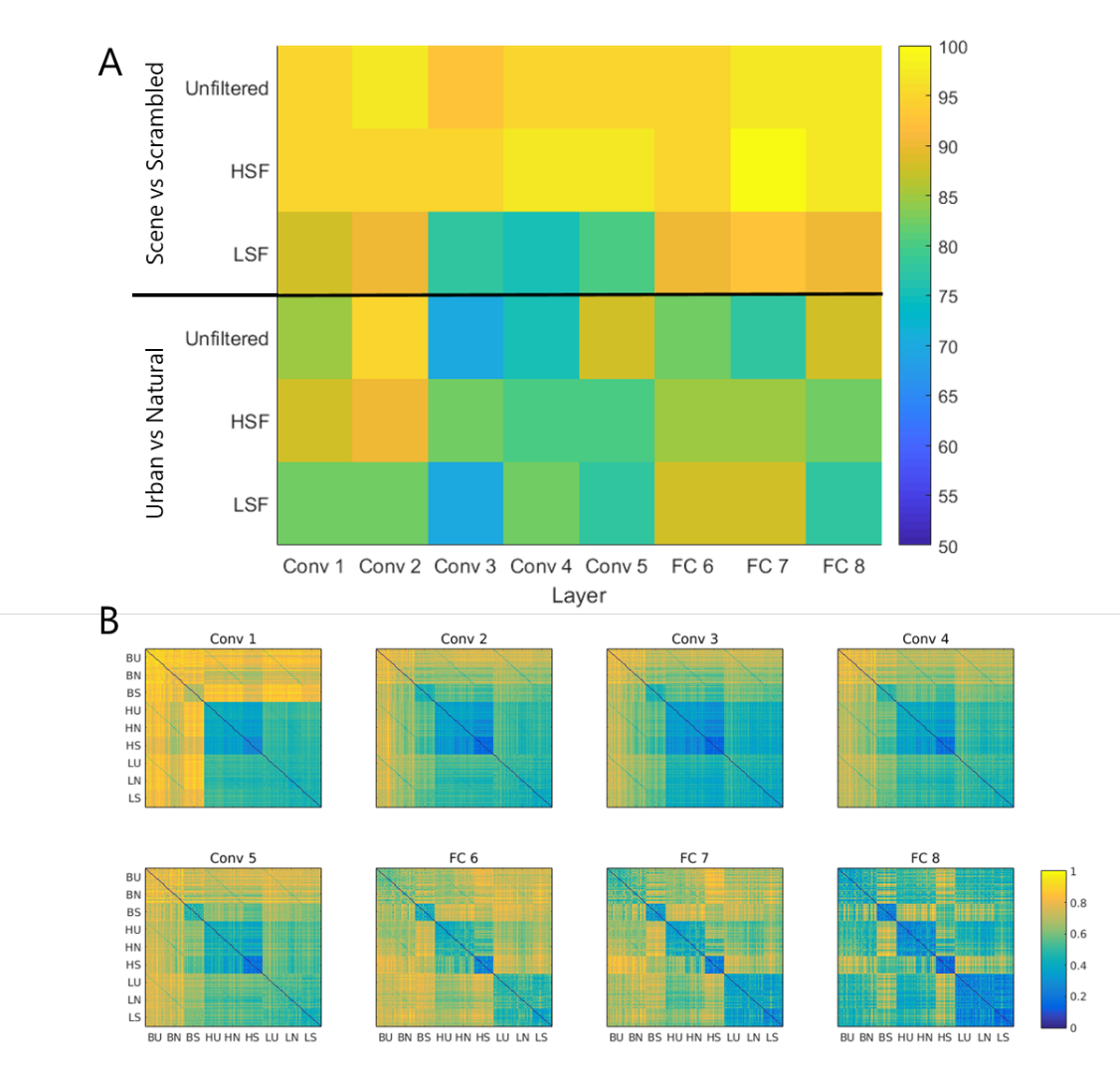


**Supplementary Figure 4.A.** Accuracy obtained by features from each of the 8 CNN layers on the two decoding problems. **B.** Model RDMs obtained from each of the 8 CNN layers. Conv: convolutional; FC: fully connected.

**Supplementary analysis 1. Differences in event-related fields (ERF)**

To test for scene-selective responses present in the event-related fields (ERF), the data were downsampled to 600 Hz, bandpass-filtered between 0.5 and 30 Hz and baseline corrected using the 500 ms time window prior to stimulus onset. Axial gradiometer ERFs were realigned to a common sensor position (Knösche, 2002) and averaged across subjects. Based on local minima in the global field power across all trials (Figure 5A), we identified three time windows of interest (Perry and Singh, 2014): 84-143 ms, 143-343 ms, and 343-401 ms. For each time window, we tested for differences between responses to unfiltered (broadband) scenes and scrambled stimuli at all MEG sensors, using paired t-tests and randomization testing (5000 iterations, corrected for multiple comparisons using the maximal statistic distribution).

The largest amplitudes in response to scenes in this dataset were found over occipital and temporal sensors (Figure 5B). Significant differences in the response to scenes and scrambled scenes were found over temporal sensors (343-401 ms; *P*<0.01, t(18)>4.72). At the P2 latency, differences were present between scenes and scrambled stimuli at two occipital sensors, but they failed to survive correction for multiple comparisons (143-343 ms; *P*>0.034, t(18)<4.4). No significant differences between scenes and scrambled scenes were found at the P1 latency (*P*>0.4, t(18)<2.81).


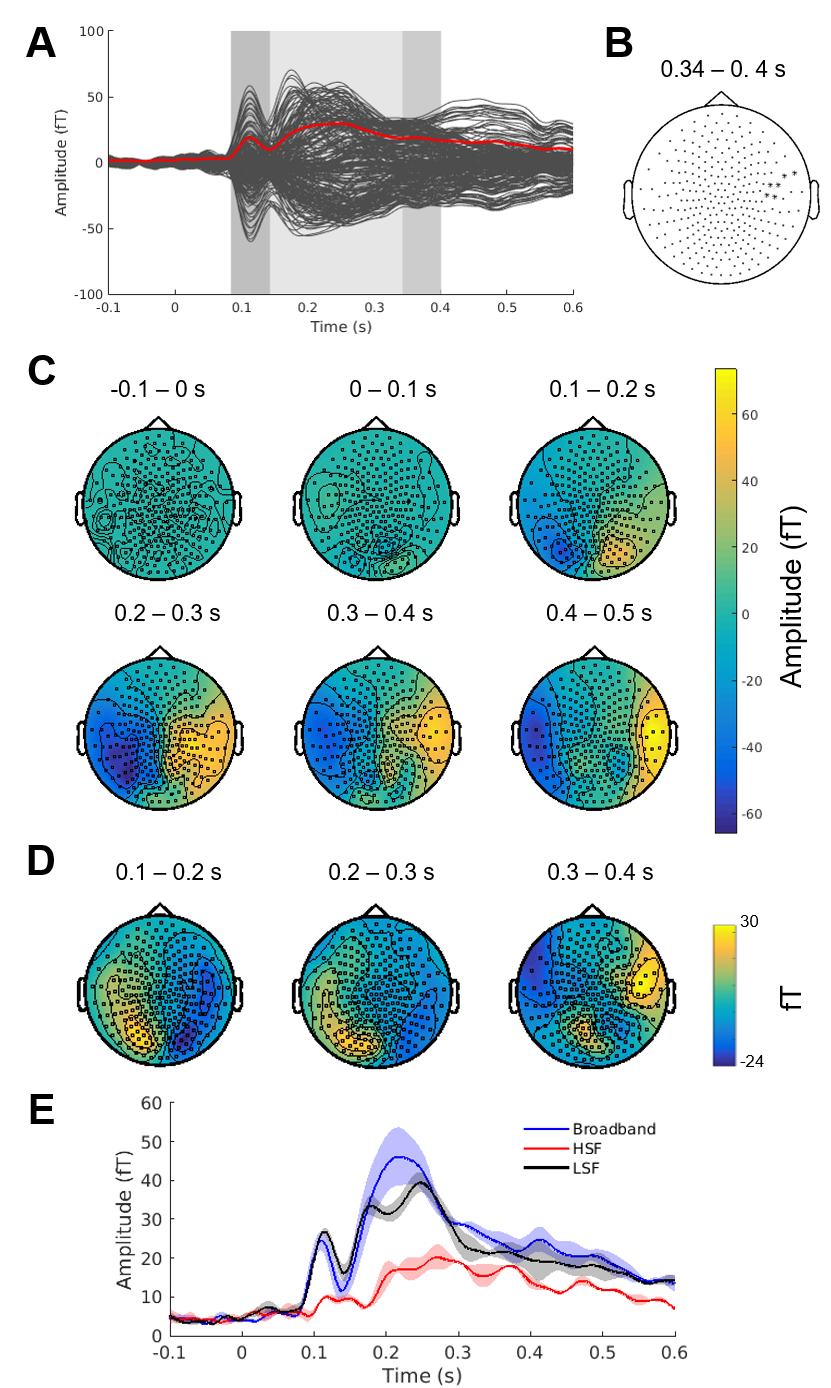


**Supplementary Figure 5. A.** Butterfly plot of amplitudes over all trials and all sensors (black) overlaid by the global field power plots for all trials (red). Local minima in the GFP plot were used to determine windows of interest in the ERF analysis (the shaded gray rectangles represent different time windows). **B.** Sensors exhibiting significant differences in the response to scenes vs scrambled scenes. **C.** Grand average ERF amplitudes in response to unfiltered scenes. **D.** Difference ERF between responses to unfiltered scenes and scrambled stimuli, based on the grand average axial gradient fields. **E.** Grand average global field power for each spatial frequency condition, showing lower amplitude responses to HSF stimuli.

**Supplementary analysis 2. Sensor-space searchlight decoding of unfiltered scene categories**

We investigated the spatiotemporal dynamics of scene processing using a searchlight analysis, whereby each MEG sensor and its neighbouring sensors, defined according to a Fieldtrip template based on the CTF 275-sensor array configuration, were entered separately into the MVPA analysis. The searchlight size thus ranged between 4 and 10 sensors (mean 7.36, SD 1.12). To increase SNR, data were averaged in groups of 3 trials (Grootswagers et al., 2017). The analysis was performed using time windows of approximately 16 ms (10 sampled time points) and stratified five-fold cross-validation (note: not across exemplars) was used to evaluate classification performance. Although the reduced number of features input to the classifier may lead to a loss in accuracy compared to the sensor set analysis, the searchlight analysis can be more informative in terms of detecting effects across sensor set boundaries.

When decoding scenes from scrambled stimuli, this analysis revealed differential processing over occipital sensors starting at ~150 ms after stimulus onset, with some temporal and parietal sensors also contributing decodable information (Supplementary Figure 6). Urban versus natural scene decoding was more transient and rose above chance at a single occipital sensor cluster (Supplementary Figure 7).

**
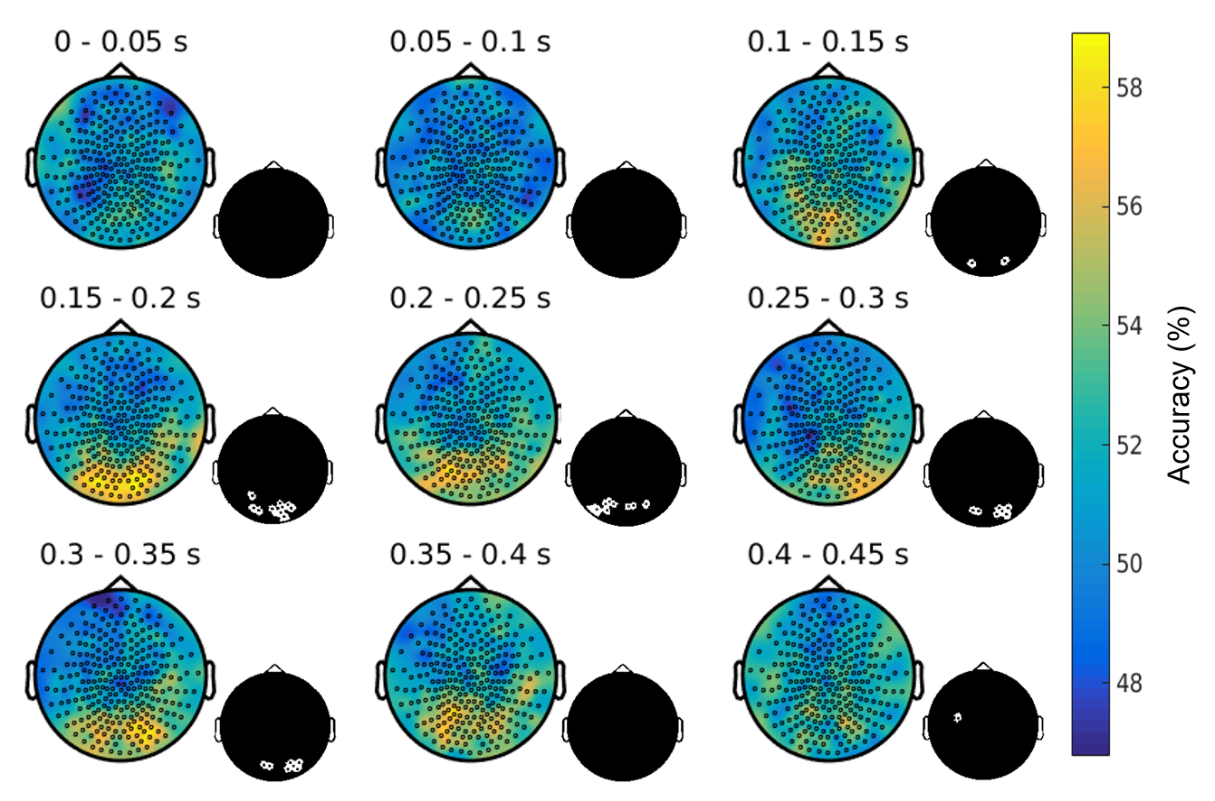
**

**Supplementary Figure 6.** Scenes vs scrambled stimuli: results of sensor-space searchlight analysis, averaged across subjects and 50 ms time windows. Main plots show decoding accuracy over time, while smaller plots show sensor clusters (in white) achieving significant decoding performance across subjects (thresholded at *P*<0.001 corrected, permutation testing).


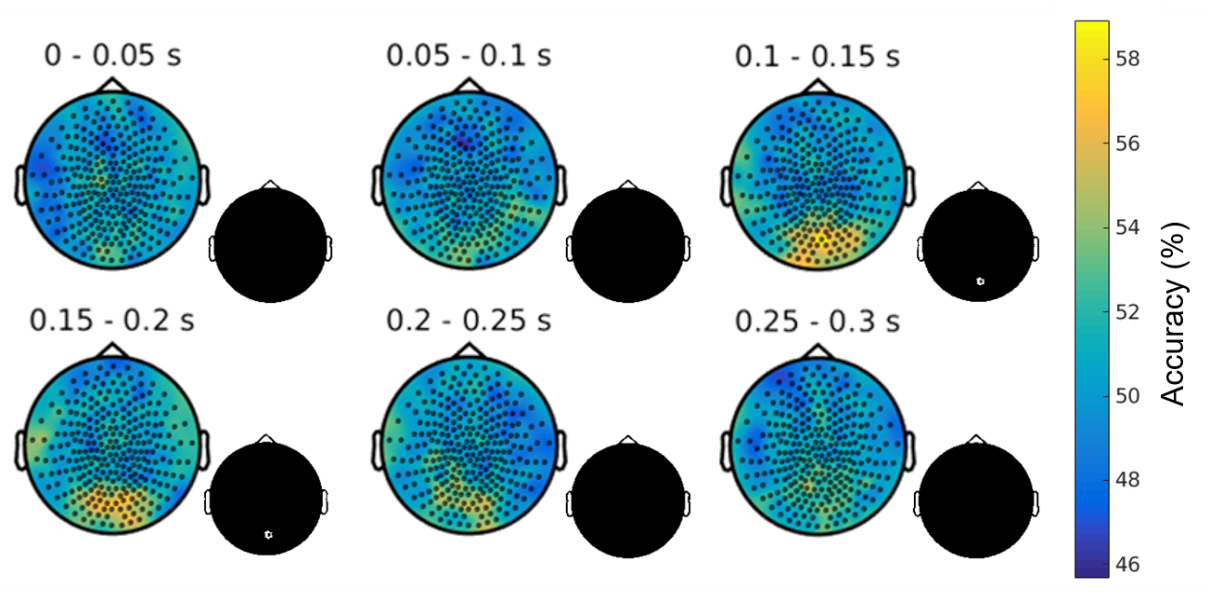


**Supplementary Figure 7.** Natural vs urban scenes: results of sensor-space searchlight analysis, averaged across subjects and 50 ms time windows. Main plots show decoding accuracy over time, while smaller plots show sensor clusters achieving significant decoding performance across subjects (thresholded at *P*<0.001 corrected, permutation testing).
